# A small molecule compound with an indole moiety inhibits the main protease of SARS-CoV-2 and blocks virus replication

**DOI:** 10.1038/s41467-021-20900-6

**Published:** 2021-01-28

**Authors:** Shin-ichiro Hattori, Nobuyo Higashi-Kuwata, Hironori Hayashi, Srinivasa Rao Allu, Jakka Raghavaiah, Haydar Bulut, Debananda Das, Brandon J. Anson, Emma K. Lendy, Yuki Takamatsu, Nobutoki Takamune, Naoki Kishimoto, Kazutaka Murayama, Kazuya Hasegawa, Mi Li, David A. Davis, Eiichi N. Kodama, Robert Yarchoan, Alexander Wlodawer, Shogo Misumi, Andrew D. Mesecar, Arun K. Ghosh, Hiroaki Mitsuya

**Affiliations:** 1 Department of Refractory Viral Infections, National Center for Global Health and Medicine Research Institute, Tokyo, Japan; 2 Department of Intelligent Network for Infection Control, Tohoku University Hospital, Miyagi, Japan; 3 Department of infectious Diseases, International Research Institute of Disaster Science, Tohoku University, Miyagi, Japan; 4 Department of Chemistry and Department of Medicinal Chemistry and Molecular Pharmacology, Purdue University, West Lafayette, IN USA; 5 Experimental Retrovirology Section, HIV and AIDS Malignancy Branch, National Cancer Institute, National Institutes of Health, Bethesda, MD USA; 6 Department of Biochemistry and Department of Biological Sciences, Purdue University, West Lafayette, IN USA; 7 Kumamoto Innovative Development Organization, Kumamoto University, Kumamoto, Japan; 8 Department of Environmental and Molecular Health Sciences, Faculty of Medical and Pharmaceutical Sciences, Kumamoto University, Kumamoto, Japan; 9 Graduate School of Biomedical Engineering, Tohoku University, Miyagi, Japan; 10 Protein Crystal Analysis Division, Japan Synchrotron Radiation Research Institute, Hyogo, Japan; 11 Protein Structure Section, Center for Structural Biology, National Cancer Institute, Frederick, MD USA; 12 Basic Science Program, Leidos Biomedical Research, Frederick National Laboratory for Cancer Research, Frederick, MD USA; 13 Viral Oncology Section, HIV and AIDS Malignancy Branch, National Cancer Institute, National Institutes of Health, Bethesda, MD USA; 14 Department of Infectious Diseases, Graduate School of Medicine and Tohoku Medical Megabank Organization, Tohoku University, Miyagi, Japan; 15 Department of Clinical Sciences, Kumamoto University Hospital, Kumamoto, Japan

**Keywords:** Antimicrobials, SARS-CoV-2

## Abstract

Except remdesivir, no specific antivirals for SARS-CoV-2 infection are currently available. Here, we characterize two small-molecule-compounds, named GRL-1720 and 5h, containing an indoline and indole moiety, respectively, which target the SARS-CoV-2 main protease (M^pro^). We use VeroE6 cell-based assays with RNA-qPCR, cytopathic assays, and immunocytochemistry and show both compounds to block the infectivity of SARS-CoV-2 with EC_50_ values of 15 ± 4 and 4.2 ± 0.7 μM for GRL-1720 and 5h, respectively. Remdesivir permitted viral breakthrough at high concentrations; however, compound 5h completely blocks SARS-CoV-2 infection in vitro without viral breakthrough or detectable cytotoxicity. Combination of 5h and remdesivir exhibits synergism against SARS-CoV-2. Additional X-ray structural analysis show that 5h forms a covalent bond with M^pro^ and makes polar interactions with multiple active site amino acid residues. The present data suggest that 5h might serve as a lead M^pro^ inhibitor for the development of therapeutics for SARS-CoV-2 infection.

## Abstract (web summary)

Here, using in vitro assays and structural analysis, the authors characterize the anti-SARS-CoV-2 properties of two small molcules, showing these to bind and target the virus main protease (M^pro^), and to exhibit a synergistic antiviral effect when combined with remdesivir in vitro.

## Introduction

The novel coronavirus disease 2019 (COVID-19) caused by a positive-strand RNA virus, severe acute respiratory syndrome coronavirus 2 (SARS-CoV-2), started in Wuhan, Hubei province, China^1–4^ and escalated into the pandemic. As of December 14, 2020, more than 71 million COVID-19 cases have been reported in 220 countries and more than 1.6 million deaths^5^. Currently, except for remdesivir, which was most recently approved as the first proven emergency therapeutic for treating COVID-19, no specific therapeutics are available. The hope that the COVID-19 pandemic subsides with “herd immunity” is likely to be disappointing. Moreover, it is not clear whether COVID-19-convalescent people with antibodies to SARS-CoV-2 are immune to reinfection. It is of utmost urgency to develop effective antivirals, therapeutics that mitigate the lethal consequences of cytokine storm and effective vaccines.

**Fig. 1:**
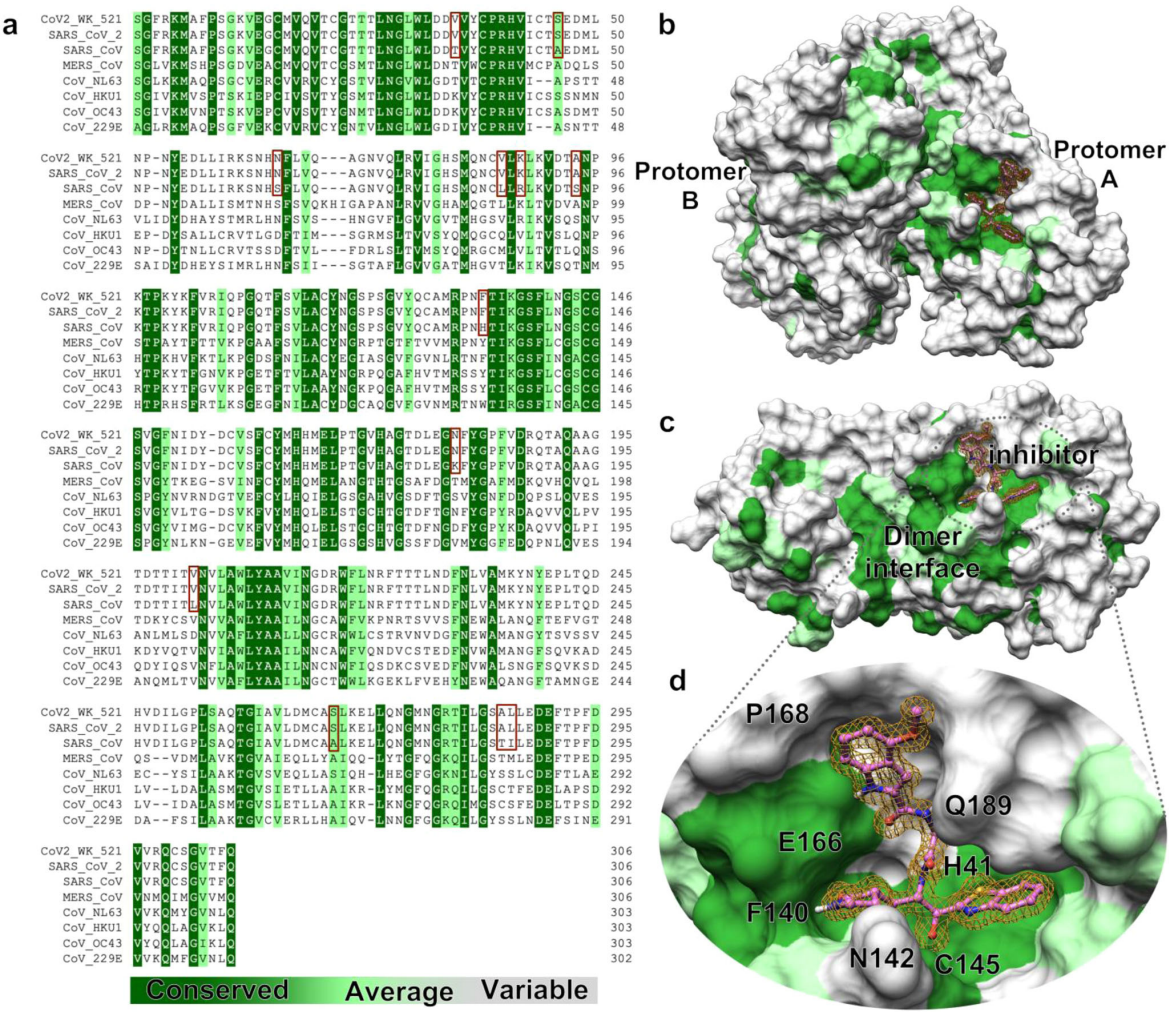
Human coronaviruses Main proteases (M^pro^s) are highly conserved around the dimer interface and ligand binding groove. **a** Amino acid sequence alignment of main proteases (M^pro^s) of eight human coronaviruses are shown. Identical amino acids are indicated in dark green; moderately conserved residues in light green and variable amino acids in light gray. The sequence of M^pro^ of SARS-CoV-2^WK-521^ used in the present study is completely identical to that of SARS-CoV-2 (PDB:6LU7) and shares 96% identity with the SARS-CoV (PDB: 2AMQ). The 12 amino acid residues that differ between the sequence of SARS-CoV’s M^pro^ and that of SARS-CoV-2^WK-521^ are highlighted in pink. Other less conserved sequences are from M^pro^s of MERS-CoV (PDB: 5C3N), CoV-NL63 (PDB: 5GWY), CoV-HKU1 (PDB: 3D23), CoV-229E (PDB: 2ZU2) and CoV-OC43 (Uniprot: P0C6X6). Sequence alignment was performed using Multiple Align Show (http://www.bioinformatics.org/SMS/multi_align.html). **b** Conservation of each residue is mapped onto the surface of M^pro^ structure based on the sequence alignment shown in (**a**). **c** While M^pro^s exhibit a high sequence conservation around the dimer interface in the center part of each protomer, the external part of each protomer shows high variability. **d** Omitted electron density (2Fo − Fc) of 5h, contoured at the 1σ level is shown inside the binding groove. 5h targets mainly a conserved site.

An efficient approach to drug discovery to a pathogenic agent includes the examination of existing compounds that are known to be active against related pathogens and the ensuing optimization of lead compounds. SARS-CoV-2, which causes COVID-19, belongs to the family of betacoronaviruses that includes SARS-CoV and MERS-CoV. The genome of SARS-CoV-2 has overall ~80% nucleotide identity with that of SARS-CoV^6^ and the main proteases (M^pro^) of these two viruses have 96% amino acid sequence identity (Fig. 1a). Superimposition of the structures of the M^pro^ of both SARS-CoV-2 and SARS-CoV shows near identity in their tertiary structures (Fig. 1b–d).

Structurally, the 5’ two-thirds of the viral genome encodes two overlapping polyproteins, pp1a and pp1ab, which are processed to generate the viral replication complex, replicase polyprotein, which undergoes processing by two viral proteases, the M^pro^ and papain-like protease (PL^pro^)^7–11^. The role of the two proteases is essential for the replication of SARS-CoV and both proteases have been recognized as attractive targets for developing antiviral agents^12–15^. Based on the high structural homology, we examined potential activity of experimental M^pro^ inhibitors, which had been shown to be active against SARS-CoV^16–19^, against a newly isolated SARS-CoV-2 strain, JPN/TY/WK-521 (SARS-CoV-2^WK-521^), in VeroE6 cell-based assay employing RNA-qPCR assay, cytopathicity assays, and immunocytochemistry. Indeed, the M^pro^ of SARS-CoV-2^WK-521^ (PDB: 6XR3) proved to have the same amino acid sequence identity with the M^pro^ of SARS-CoV-2 (PDB:6LU7) (Fig. 1a). Here, we demonstrated that two compounds, GRL-1720 and compound 5h^20^, containing an indoline and indole moiety, respectively, potently block the infectivity of SARS-CoV-2 by targeting M^pro^. Importantly, 5h blocked the infectivity and cytopathicity of the virus with high potency and without any detectable cytotoxicity even at 200 µM, as examined using detailed immunocytochemistry.

## Results

### GRL-1720 and compound 5h potently block the infectivity and cytopathicity of SARS-CoV-2^WK-521^

**Fig. 2:**
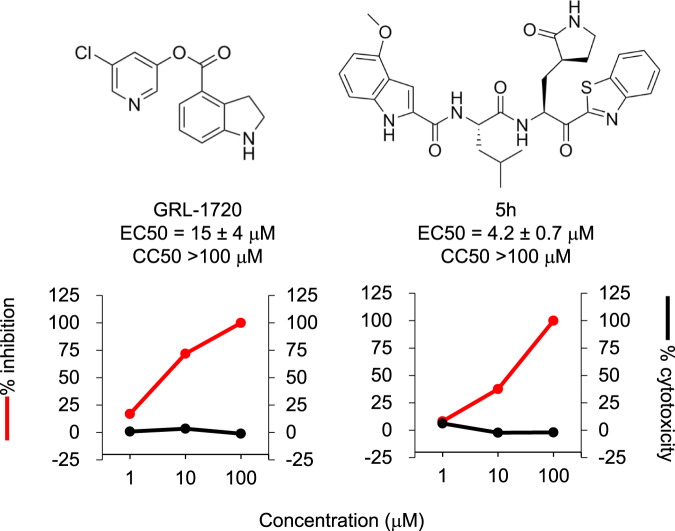
The antiviral activity of GRL-1720 and compound 5h against SARS-CoV-2. VeroE6 cells were exposed to SARS-CoV^WK-521^ for 1 h, the virus was washed out, and the virus-exposed VeroE6 cells were cultured for 3 days. The viral copy numbers in the culture supernatants were determined using RT-qPCR. Red and black lines indicate reduction of viral copy numbers and cytotoxicity, respectively. Each compound was tested upon at least three different occasions. The data in Fig. 2 are representative ones. Both graphs were generated with Microsoft Excel. EC_50_ values shown denote means ± 1 S.D. All compounds shown in Fig. 2 were tested and compared in one assay performed in duplicate (*n* = 2). Source data are provided as a Source Data file.

We have examined a variety of compounds that have reportedly been active against SARS-CoV^21^. We specifically selected and synthesized a panel of active compounds based on previously known structures and their activity against SARS-CoV^16–20^. We demonstrated previously that two compounds, GRL-1720 (Supplementary Fig. 1) and 5h (Supplementary Fig. 2), potently inhibit M^pro^ from SARS-CoV and have anti-SARS-CoV activity^16,20^. They also potently block the enzymatic activity of SARS-CoV-2 M^pro^ and the infectivity, replication, and cytopathicity of SARS-CoV-2^WK-521^ (Fig. 2 and Supplementary Fig. 3). GRL-1720 and 5h potently inhibited the enzymatic activity of SARS-CoV-2 M^pro^. GRL-1720 is an irreversible, covalent inhibitor of SARS-CoV-2 M^pro^ with time-dependent inhibition kinetic parameters of *k*_*inact*_ = 2.53 ± 0.27 min^−1^, K_i_ = 2.15 ± 0.49 μM and a second-order rate constant, *k*_*inact*_/K_i_ = 19,610 M^−1^ ± 4,930 sec^−1^ (Supplementary Fig. 3a, b). The IC_50_ value for GRL-1720 after a 10 min incubation is 0.32 ± 0.02 μM (Supplementary Fig. 3c). 5h on the other hand is a tight-binding, reversible-covalent inhibitor of SARS-CoV-2 M^pro^ with a K_i_ value of 17.6 ± 3.2 nM as determined using the Morrison equation (Supplementary Fig. 3d). As assessed using the quantitative VeroE6 cell-based assay with RNA-qPCR, the EC_50_ values of GRL-1720 and compound 5h were 15 ± 4 and 4.2 ± 0.7 μM, respectively, and apparent CC_50_ values were both >100 μM (Fig. 2).

**Fig. 3:**
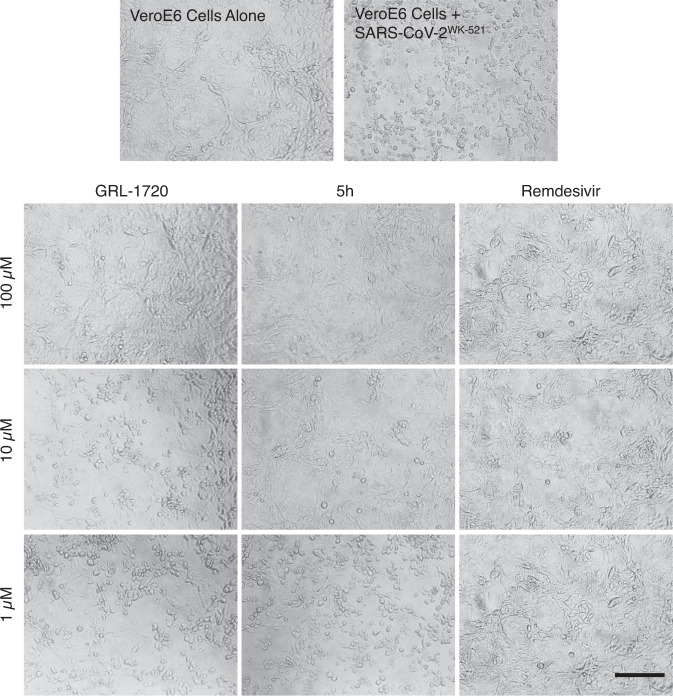
GRL-1720 and 5h exert potent activity against SARS-CoV-2^WK-521^. VeroE6 cells were exposed to SARS-CoV-2^WK-521^ for 1 h, the virus was washed off, and the cells were cultured in the presence of various concentrations of test compound for 3 days. The images shown are those captured under light microscopy. VeroE6 cells cultured alone (top left) appear to be robust; however, those exposed to SARS-CoV-2^WK-521^ and cultured in the absence of test compounds, a number of cells were destroyed by the cytopathicity of the virus and appear granular (top right). VeroE6 cells exposed to the virus and cultured with each of the two compounds, GRL-1720 and 5h (at 1, 10 and 100 µM). The virus-exposed cells cultured with 100 µM, all the cells appeared to be completely protected. Representative images from three independent experiments are shown. Scale bar = 200 µm.

It is noteworthy that cell cytotoxicity of certain compounds reduces virion production by the cells and such reduction is often misinterpreted as antiviral activity of the compounds. For example, the anti-cancer/leukemia agent, daunorubicin (*aka* Adriamycin), was reported to be active against HIV-1^22,23^, but that anti-cancer/leukemia agent never proved to be of clinical utility as an anti-HIV-1 agent. Thus, we carefully asked if the three compounds, GRL-1720, 5h, and remdesivir exerted cytotoxicity at 1, 10, and 100 μM. As seen in Fig. 3, VeroE6 cells cultured alone (top left in Fig. 3) appeared to be robust and had spread at the bottom of the microtiter culture plates; however, when VeroE6 cells were exposed to SARS-CoV-2^WK-521^ and cultured in the absence of test compounds, almost all the cells acquired granular patterns and became detached from the bottom of the culture plate (top right in Fig. 3), indicating the cells were infected and killed by the cytopathicity of the virus. In contrast, when the SARS-CoV-2^WK-521^-exposed cells were cultured in the presence of each of the three compounds (GRL-1720, 5h, and remdesivir) at 10 μM, the cells appeared to be moderately protected, and at 100 μM, all the cells appeared to be completely protected by each compound. All three images of the cells cultured with 100 μM of each compound appeared to be similar to the image of the cells cultured without the virus (top left in Fig. 3). Moreover, 5h, at 200 μM, showed no significant cytotoxicity in a human lung cancer cell line, Calu-3, or two human primary cells [peripheral blood mononuclear cells (PBMC) and human bronchial/tracheal epithelial cells (HBTEC)]. In contrast, remdesivir showed cytotoxicity at 200 μM and its CC_50_ value was 138 ± 9 μM in HBTEC (Supplementary Table 1). When primary human airway epithelial cells were cultured in the air-liquid interface (ALI) setting in the presence of up to 100 μM of 5h and remdesivir, no detectable cytotoxicity was observed, although those cells were poorly susceptible to the infectivity of SARS-CoV-2^WK-521^ (Supplementary Fig. 4). These results strongly suggest that 5h exerts its potent inhibitory activity against SARS-CoV-2 at concentrations that do not bring about detectable cytotoxicity.

### GRL-1720 and 5h are potent against SARS-CoV-2^WK-521^ as assessed with immunocytochemistry

**Fig. 4:**
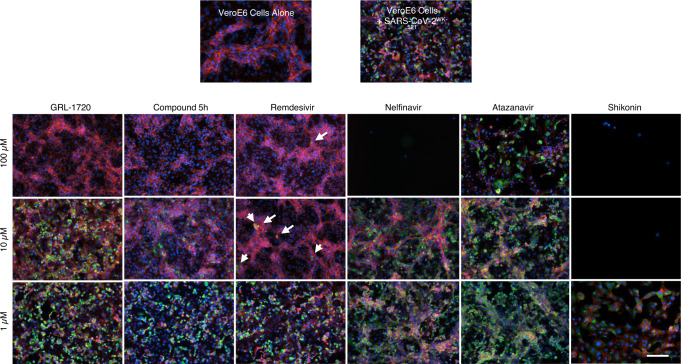
GRL-1720 and 5h exert potent activity against SARS-CoV-2^WK-521^ but shikonin, nelfinavir, and atazanavir, reportedly active against SARS-CoV-2, failed to block the infection by SARS-CoV-2^WK-521^. VeroE6 cells were exposed to SARS-CoV-2^WK-521^ for 1 h, the viruses were washed out, and the virus-exposed cells were cultured in the presence or absence of various concentrations of each test compound for 3 days. Immunocytochemistry was then performed using an IgG fraction prepared from COVID-19-convalescent plasma as a primary antibody. Viral breakthrough was observed in the cells cultured in the presence of remdesivir at 100 µM, while in the cells cultured with 100 µM GRL-1720 and 5h, no such virus breakthrough was found. Arrows denote the cells infected by the virus in the presence of high concentrations of remdesivir. Such breakthrough cells (stained in green) are often seen in clusters. SARS-CoV-2 antigens, F-actin, and nuclei are stained in green, red, and blue, respectively. Representative images from three independently conducted experiments are shown. Scale bar = 100 µm.

In order to confirm and corroborate the potent antiviral activity of GRL-1720 and 5h observed using the quantitative RNA-qPCR assay, which often does not differentiate the actual antiviral activity from the misleading and distractive apparent “antiviral effect” caused by the cytostatic effect and/or cytotoxicity of test compounds, we employed immunocytochemistry, which can allow us to examine the antiviral activity of test compounds at the cellular level. For the primary antibody in our immunocytochemistry, we used an IgG fraction isolated from a COVID-19-convalescent patient, who proved to have high titers of neutralizing antibodies as well as SARS-CoV-2-binding IgG antibodies (Data not shown). As shown in Fig. 4, when VeroE6 cells were cultured alone, robust cellular cytoskeleton filamentous actin (F-actin) was seen as mesh-like structures (shown in red) and a number of nuclei (shown in blue) were identified, signifying that those cells were healthy and replicating (top left in Fig. 4). However, when VeroE6 cells were exposed to SARS-CoV-2^WK-521^ and cultured in the absence of test compound, the F-actin structure was lost and a number of cells were infected by the virus (stained in green; top right in Fig. 4). In contrast, in SARS-CoV-2^WK-521^-exposed VeroE6 cells that were cultured in the presence of 100 μM GRL-1720, there was a significant reduction in the number of SARS-CoV-2^WK-521^-infected cells and there were essentially no infected cells when the cells were cultured in the presence of 100 μM GRL-1720. In contrast, in the presence of as low as 10 μM 5h, there was significant reduction in the number of infected cells and there were no infected cells when VeroE6 cells were cultured at 100 μM 5h. Remdesivir also significantly reduced the number of infected cells at 10 and 100 μM; however, there was viral breakthrough in the culture and some infected cells were identified. The viral breakthrough in VeroE6 cell culture in the presence of remdesivir is shown in higher magnification in Supplementary Fig. 5.

In order to further examine the antiviral activity of remdesivir, GRL-1720, and 5h, we tested the antiviral activity in SARS-CoV-2^WK-521^-exposed VeroE6 cells that were cultured in the presence of a wide range of concentrations of each compound. In the set of immunocytochemistry data, there was no virus breakthrough in all of the fields examined using Cell Imaging Multi-Mode Reader (Cytation 5) at concentrations 50 through 100 μM; however, there were obvious virus breakthroughs at 150 and 200 μM of remdesivir (Supplementary Fig. 6). In contrast, there was no virus breakthrough identified in the cells cultured in the presence of 5h at concentrations ranging 20–200 μM (Supplementary Fig. 6). Of note, while the amounts of F-actin appeared to have decreased at 100 μM and higher concentrations of remdesivir, there was no significant decrease in the amounts of F-actin throughout the wide range of concentrations of 5h tested, suggesting that 5h is less cytotoxic than remdesivir (Supplementary Fig. 6 and Supplementary Table 1).

Shikonin was reported by Jin et al.^24^ to be well-docked into the putative hydrophobic cavity of M^pro^’s active site and shown by that group to exert activity against SARS-CoV-2; however, shikonin totally failed to block the infectivity of SARS-CoV-2^WK-521^ and was highly toxic even at 1 μM (VeroE6 cells were swollen and apparently dying). At 10 and 100 μM, all the cells were presumably killed by the direct toxicity of shikonin as examined with detailed immunocytochemistry (Fig. 4). Two HIV-1 protease inhibitors, nelfinavir and atazanavir, which have also been reported to be active against SARS-CoV-2^25–27^, did not show any detectable anti-SARS-CoV-2 activity and, in our study, nelfinavir was highly toxic at 100 μM (Fig. 4). These data strongly suggest that the apparent anti-SARS-CoV-2 effects of various compounds have been mistakenly interpreted as activity against the virus and that the cytostatic and cytotoxicity of such compounds are to be carefully examined.

### Compound 5h as combined with remdesivir exerts synergistic activity against SARS-CoV-2 and viral breakthrough does not occur

**Fig. 5:**
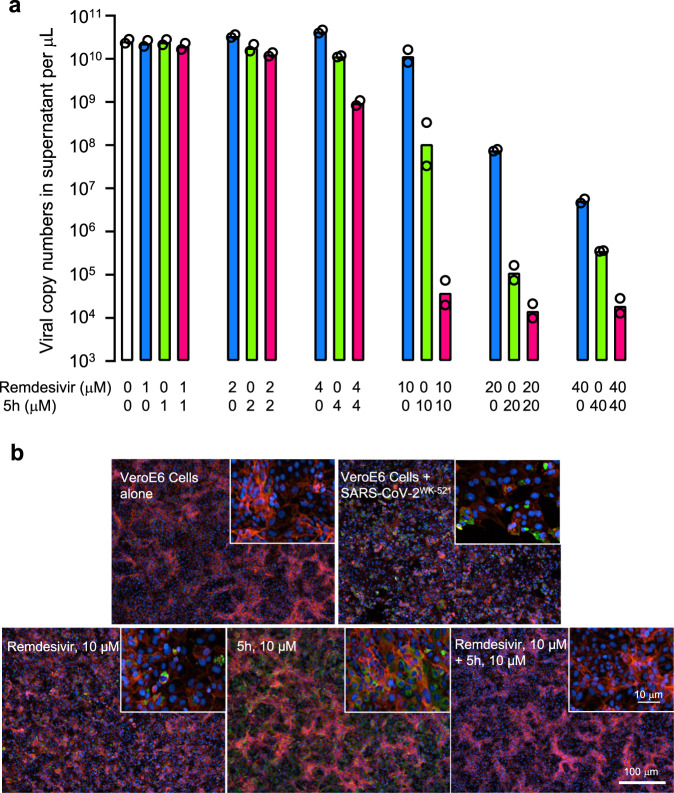
Compound 5h combined with remdesivir does not permit viral breakthrough. VeroE6 cells were exposed with SARS-CoV-2^WK-521^ for 1 h, the virus was washed out, and the virus-exposed cells were cultured for 3 days in the presence of compounds. **a** Viral RNA copy numbers in the culture supernatants were determined using RNA-qPCR. The numbers of viral RNA copy were more significantly reduced in the combination of 5h and remdesivir (each at 4, 10, 20, and 40 µM) than 5h or remdesivir alone. The mean EC_50_ values of 5h and remdesivir determined with this set of the data were 4.0 and 9.0 µM, respectively. The figure was generated with GraphPad Prism 9. Bars indicate geometric mean (*n* = 2). **b** Representative images of fluorescent immunocytochemistry data from two independently conducted experiments are shown. SARS-CoV-2 antigens, F-actin, and nuclei are indicated in green, red, and blue, respectively. Source data are provided as a Source Data file.

Remdesivir is a nucleotide analog that reportedly blocks the infectivity of SARS-CoV-2 through acting as an inhibitor of a viral RNA-dependent RNA polymerase (RdRp)^28,29^, whereas 5h blocks the infectivity of the virus through acting as an M^pro^ inhibitor. Thus, we asked whether these two compounds work against SARS-CoV-2 in an additive or synergistic fashion in the VeroE6 cell-based assays (Fig. 5). The cells were exposed to SARS-CoV-2^WK-521^ and cultured in the presence or absence of various concentrations of remdesivir in combination with various concentrations of 5h. Remdesivir (2 μM) and compound 5h (2 μM) suppressed the viral replication by 0.67-fold (the geometric mean of viral RNA copy number was 3.4 × 10^10^ with a range 3.1-3.6 × 10^10^ copies/μL) and 1.3-fold (1.8 × 10^10^; 1.5-2.2 × 10^10^), respectively, while when the two compounds were combined, the suppression was by 1.8-fold (1.3 × 10^10^; 1.2-1.4 × 10^10^). Remdesivir (4 μM) and 5h (4 μM) suppressed the viral replication by 0.53-fold (4.3 × 10^10^; 3.9-4.7 × 10^10^) and 2.0-fold (1.1 × 10^10^; 1.1-1.2 × 10^10^), respectively, while when combined, the suppression was by 24-fold (9.5 × 10^8^; 8.3 × 10^8^-1.1 × 10^9^). At 10 μM, remdesivir and 5h suppressed by 20-fold (1.2 × 10^10^; 8.3 × 10^9^-1.6 × 10^10^) and 210-fold (1.1 × 10^8^; 3.3 × 10^7^-3.3 × 10^8^), respectively, while, when combined, the suppression was by 590,000-fold (3.8 × 10^4^; 2.0-7.4 × 10^4^). The apparent combination effect was maximal when 20 μM remdesivir and 20 μM 5h were combined, producing the suppression by as much as 1,600,000-fold (1.4 × 10^4^; 9.9 × 10^3^-2.1 × 10^4^)(Fig. 5a and Supplementary Table 2). The use of 40 μM did not further the suppression, suggesting that 20 μM combination of the two compounds exerted the maximal suppression of the viral replication. When examined using the Bliss additivism method that tests the presence or absence of synergism, additivism or antagonism^30–32^, the reduction achieved by combinations of the two agents was synergistic (Supplementary Table 2).

We also examined whether the synergistic effects of the combination were seen using immunocytochemistry. As seen in Fig. 5b, at 10 μM, both remdesivir alone and 5h alone failed to completely block the infectivity of SARS-CoV-2^WK-521^ and a number of cells were infected and stained green. However, when these two compounds were combined, the cells were completely protected from viral infection (Fig. 5b), in line with the data shown in Fig. 5a. At 20 μM, in the presence of remdesivir or 5h, there was significant virus breakthrough; however, when combined, there was no breakthrough detected at all. The same was true when the two compounds were examined at 40 μM (Supplementary Fig. 7 and Supplementary Table 2).

### Molecular interactions of 5h and GRL-1720 with SARS-CoV-2’s M^pro^

**Fig. 6:**
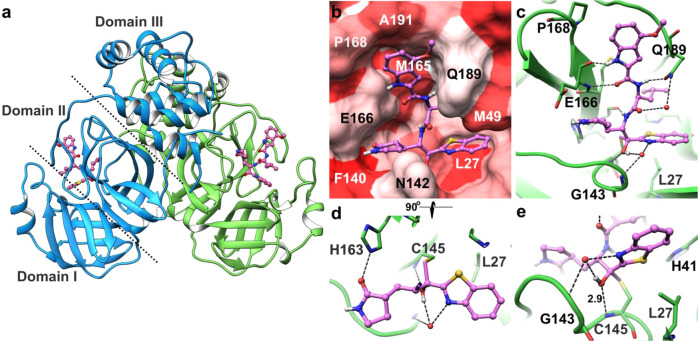
The X-ray crystal structure of SARS-CoV-2 M^pro^ in complex with 5h. **a** SARS-CoV-2 M^pro^ is shown in ribbon and 5h in ball and stick representation. The two protomers of M^pro^ are shown in green and blue ribbons, and 5h in pink. Nitrogen, oxygen, and sulfur atoms are shown in blue, red and yellow, respectively. Each protomer consists of three domains (only one protomer is labeled for clarity) and domains I and II mostly comprise of b-sheets and loops and forming the binding pocket. **b** Hydrophobicity of the binding pocket is represented by the intensities of red color, hydrophobic residues such as Leu-27 and Met-165 are shown in dark red, whereas polar or charged residues such as Glu-166, Gln-189 are shown in light red. While the distal edge of 4-methoxyindole-2-carbonyl group is surrounded by Pro-168 and Ala-191 on the top, the center part of the moiety faces to Met-165, which lies in the interior part of the binding groove. **c** Hydrogen bond interactions between 5h (pink carbon atoms) and M^pro^ (green carbon atoms) are shown in black dashed lines. 5h forms 8 direct hydrogen bonds with M^pro^ residues, additional polar interactions are mediated by water molecules (red spheres). **d** A 90°-rotated view of 5h focuses on the interactions of 2-oxopyrrolidine and benzothiazole groups. While the nitrogen of 2-oxopyrrolidine forms hydrogen bonds with the carboxylate oxygen of Glu-166 and carbonyl oxygen of Phe-140, the oxygen of 2-oxopyrrolidine forms a hydrogen bond with the imidazole group of His-163. The nitrogen of the P1′ benzothiazole forms water-mediated hydrogen bonds with the backbone NH of Gly-143 and carbonyl oxygen of Thr-26. **e** The hydroxyl group of 5h is shown in the center, which forms a strong hydrogen bond with the backbone amide of Cys-145 (2.9 Å). Distances between atoms are shown in Å.

To understand the molecular basis of the inhibition of SARS-CoV-2’s M^pro^ by 5h, we determined the X-ray structure of M^pro^ in complex with 5h at 1.25 Å resolution. The crystallographic asymmetric unit comprises a dimer form of M^pro^. The dimer interface is highly conserved among the eight human coronaviruses M^pro^s listed in Fig. 1a. Indeed, a number of previous studies reported that only the dimeric form of M^pro^ shows enzymatic activity (Fig. 1b-d)^33,34^. In fact, when we have gone over the amino acid sequences of M^pro^ of 102 different SARS-CoV-2 strains isolated in 12 nations and regions, whose full-length viral sequences were deposited to GenBank at National Center of Biotechnology Information (NCBI) by March 17, 2020, all strains proved to carry an identical M^pro^ amino acid sequence to the M^pro^ of NCBI Reference Sequence, NC_045512.2 (https://www.ncbi.nlm.nih.gov/nuccore/NC_045512) and the M^pro^ of SARS-CoV-2 (PDB:6LU7). In the X-ray crystallographic data collected, clear electron density of 5h (Supplementary Fig. 8) was observed inside the binding groove stretching between domain I and domain II (Fig. 6a). Detailed molecular interactions with M^pro^ are shown in the Fig. 6b–e. 5h fully occupies all binding pockets and is stabilized by six direct hydrogen bonds with the residues inside the binding groove of M^pro^. Particularly, Glu-166 engages in the formation of two hydrogen bonds linking the main chain carbonyl and the amide group of Glu-166. In the central part of 5h, additional hydrogen bonds form with the side-chain oxygen of Gln-189 and the main chain carbonyl of His-164 (Fig. 6b, d). Since the majority of hydrogen bonds form through the main chain carbonyl and NH groups of M^pro^, those interactions are less likely affected by potential mutations. In addition to hydrogen bonds, several hydrophobic residues contribute to binding affinity via van der Waals interactions (Fig. 6c). Inside the S2 sub-pocket, the distal benzene ring of the benzothiazole of 5h is sandwiched by Leu-27 and Met-49. Overall, the chemical composition of 5h matches well with the surface of the binding groove in terms of the hydrophobicity scale. The observed continuous electron density of the tetrahedral ketal and the sulfur atom of Cys-145 indicates the formation of a covalent bond between 5h and M^pro^ (Fig. 6e, supplementary Fig. 8). The sulfur atom of Cys-145 undergoes nucleophilic addition reaction and forms a covalent bond with the carbonyl carbon (-C=O) next to the benzothiazole of 5h, resulting in the conversion of the carbonyl to an alcohol (-C-OH) and to the formation of one direct hydrogen bond and water-mediated hydrogen bond interactions around the three oxyanion hole residues, Cys-145 and Gly-143 (Fig. 6e). Unlike in most structures of the free and inhibited SARS-CoV-2 M^pro^ (Supplementary Fig. 9), the “catalytic water”, bound by His-41, His-164, Asp-187, and the main chain amide nitrogen of His-41, is not present in the structure of the 5h complex. This significant modification of the active site is caused by the shift of the side chain of His-41 due to its interaction with the benzothiazole of the inhibitor, creating a direct hydrogen bond between the imidazole groups of His-41 and His-164.

GRL-1720 is an indoline chloropyridinyl ester. The mechanism of interaction of esters with SARS-CoV has been examined by others^35^. We propose a similar mechanism for the interactions of GRL-1720 with SARS-CoV-2 M^pro^, which is outlined in Supplementary Fig. 10. The catalytic His-41 and Cys-145 are involved in the nucleophilic attack on the ester carbon of GRL-1720. Following acylation, the chloropyridinyl group departs, and the carbonyl indoline moiety is bound to Cys-145 of M^pro^ through a covalent bond (indicated by a yellow arrow in Supplementary Fig. 11). The presence of a covalently-bound carbonyl indoline moiety was verified by electrospray ionization quadrupole time-of-flight mass spectrometry (ESI-QTOF/MS)(*vide infra*). The predicted structural interactions of the carbonyl indoline moiety of GRL-1720 with M^pro^ are shown in Supplementary Fig. 11. The carbonyl carbon is involved in hydrogen-bond interactions with the backbone amine nitrogens of Gly-143 and Cys-145 (Supplementary Fig. 11). Overall, 5h has multiple additional favorable interactions with M^pro^ compared with that of GRL-1720, which may partially explain the much greater enzymatic inhibition and antiviral activity of 5h compared to GRL-1720.

### Analyses of interactions of GRL-1720 and 5h using nanoLC-ESI-QTOF-MS

**Fig. 7:**
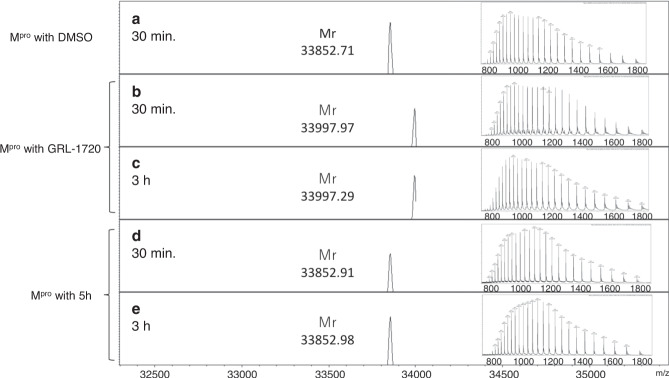
Mass spectrometry analysis of apoM^pro^, GRL-1720- and 5h-treated Mpro. Deconvoluted mass spectra of 10 µM apoM^pro^ treated with DMSO (**a**), GRL-1720 (**b** and **c**) and 5h (**d** and **e**) are shown for 30 min and 3 h, respectively. The insets show the molecular ion profiles for apoM^pro^, and GRL-1720- and 5h-treated M^pro^. The theoretical molecular mass is 33,853.41 Da (average mass) for apoM^pro^ and 33,998.56 Da (average mass) for the 1*H*-indpline-4-carbonyl group-binding form of M^pro^.

In order to ask whether GRL-1720 and 5h form covalent bonds, nanoLC-ESI-QTOF-MS experiments were performed to interrogate the nature of the interactions after treatment of M^pro^ with GRL-1720 or 5h for 30 min and 3 h. As compared with the mass of apoM^pro^ (Fig. 7a for 30 min treatment), the mass increments of +145.26 Da (Fig. 7b for 30 min treatment) and +144.58 Da (Fig. 7c for 3 h treatment) in the presence of GRL-1720, were observed. The increased size corresponds to the molecular weight of the bound 1*H*-indoline-4-carbonyl group. In the active site of M^pro^, Cys-145 and His-41 appear to form a catalytic dyad (Supplementary Fig 10). Consequently, the sulfur atom gains nucleophilicity, and presumably exerts a nucleophilic attack on the electrophilic carbon atom of the polar carbonyl group from GRL-1720. In contrast, no changes in the mass were identified in M^pro^ treated with 5h for 30 min or 3 h as compared with M^pro^ unexposed to 5h (Fig. 7d, e). These data suggest that 5h does form a covalent bond with M^pro^ as observed in the X-ray crystallographic analyses (Fig. 6); however, the covalent bonding is of a reversible nature as previously described by Vershueren et al.^35^.

### Thermal stability of M^pro^ in the absence or presence of GRL-1720 and 5h

**Fig. 8:**
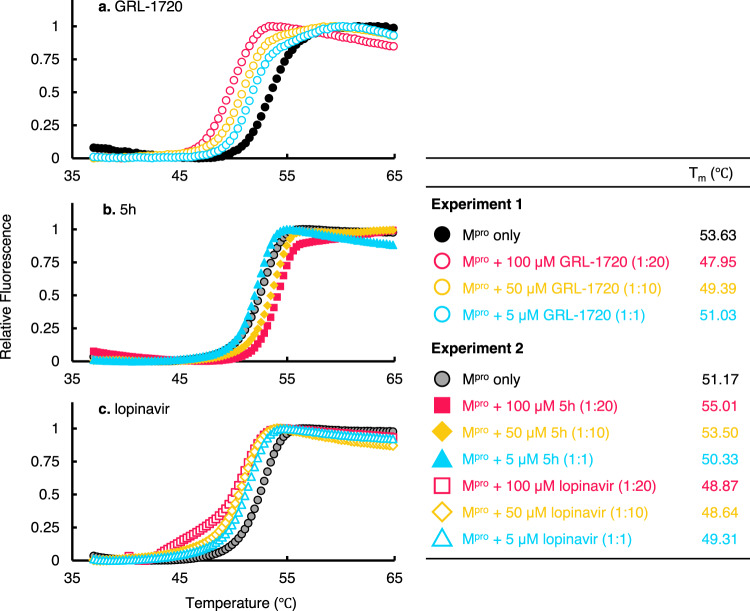
Thermal stability of M^pro^ in the presence or absence of 5h. Thermal stability of M^pro^ in the presence of GRL-1720 (**a**), 5h (**b**), and lopinavir (**c**) using differential scanning fluorimetry (DSF) was determined. Note in Experiment 1 that the T_m_ value of M^pro^ alone was 53.63 °C, while in the presence of 50 and 100 µM of GRL-1720, the values decreased to 49.39 and 47.95 °C, respectively, suggesting that the thermal stability of M^pro^ decreased when GRL-1720 bound to M^pro^, suggesting that GRL-1720 formed covalent bond with M^pro^. In experiment 2, the T_m_ value of M^pro^ alone was 51.17 °C, while in the presence of 50 and 100 µM of 5h, the values increased to 53.50 and 55.01 °C, respectively, suggesting that the thermal stability of M^pro^ increased when 5h bound to M^pro^. When M^pro^ was treated with 50 and 100 µM lopinavir, decrement was seen with the T_m_ values with 48.64 and 48.87, suggesting that lopinavir non-specifically bound to M^pro^ (see the Results section). These data with the x-ray crystallographic analyses 5h strongly interacts with M^pro^ and potently inhibits its enzymatic activity as compared to GRL-1720. All the figures were generated with Microsoft Excel.

We also examined the thermal stability of M^pro^ in the presence of GRL-1720 and 5h using differential scanning fluorimetry (DSF). As illustrated in Fig. 8, the T_m_ value of M^pro^ alone in Experiment 1 was 53.63 °C, while in the presence of 5, 50, and 100 μM of GRL-1720, the values decreased to 51.03, 49.39, and 47.95 °C, respectively (Fig. 8a). The observed shifts of T_m_ values to lower temperatures have reportedly been associated with an apparent destabilization of the protein by covalently-bound compounds^36–38^. Thus, these data corroborate that GRL-1720 forms a covalent bond with M^pro^. By contrast, the T_m_ value of M^pro^ alone in Experiment 2 was 51.17 °C, while in the presence of 50 and 100 μM of 5h, the values increased to 53.50 and 55.01 °C (Fig. 8b). Interestingly, in the presence of 5, 50, and 100 μM of lopinavir, the T_m_ values of M^pro^ turned out to be substantially lower at 49.31, 48.64 °C and 48.87 °C (Fig. 8c), suggesting that lopinavir does not stabilize M^pro^ but rather destabilizes the protein. Of note, the interpretation of a decreased T_m_ in the case of lopinavir is complex. In general, destabilizers have been largely dismissed and removed from detailed investigations; however, all such destabilizers have been summarily placed into the non-specific binder category^36–38^. In conclusion, these thermal stability data suggest that GRL-1720 forms covalent interactions with M^pro^, while 5h likely forms reversible covalent interactions^35^ with M^pro^. Based on the X-ray crystallographic analyses and thermal stability data, it should be reasonable to conclude that 5h strongly interacts with M^pro^ and potently inhibits its enzymatic activity as compared to GRL-1720.

## Discussion

In the present study, we synthesized a panel of compounds based on previously known structures and their inhibitory activity against SARS-CoV and demonstrated that two small molecule compounds, GRL-1720^16^ and compound 5h^20^. Both compounds target the main protease (M^pro^) of SARS-CoV-2 and potently block the infectivity, replication, and cytopathicity of SARS-CoV-2^WK-521^ as assessed using cell-based assays with RNA-qPCR, cytopathicity assays, and immunocytochemistry (Figs. 2 and 3). At a concentration of 20 μM, 5h completely blocked the infection by SARS-CoV-2, while remdesivir, currently the only FDA-approved emergency therapeutic for treating patients with COVID-19^39^, permitted viral breakthrough at the same concentration. However, when 20 μM 5h was combined with 20 μM remdesivir, that combination reduced the number of SARS-CoV-2 copies by the magnitude of 1.6 × 10^6^ (Fig. 5), strongly suggesting that as in the case of antiretroviral therapy of HIV-1 infection, in which the use of one or two reverse transcriptase inhibitors and an HIV-1 protease or integrase inhibitor resulted in highly favorable antiretroviral effects^40^, such a combination might give much more favorable efficacy than remdesivir alone or 5h alone. Of particular note, in the current study, we were able to sever the reduction of numbers of virus copy numbers due to the cytostatic/cytotoxic effects from virus-specific inhibitory activity of the test compounds. Our detailed immunocytochemistry experiments clearly segregated the virus-specific antiviral activity from the cytostatic/cytotoxic effects of the test compounds. We therefore conclude that no detectable anti-SARS-CoV-2 activity is present in compounds such as HIV-1 protease inhibitors (nelfinavir and atazanavir)^25–27^ and shikonin^24^ that have previously been reportedly to be active against SARS-CoV-2. Thus far, over 80 SARS-CoV-2 M^pro^ structures in the PDB  and some of them have the authentic sequence of the N terminus, and some do not. A detailed comparison of all these structures we conducted indicated that the nature of the N terminus and its disorder bear no relationship to the mode of binding of the inhibitors. An additional structure of the 5h inhibitor complex in a different crystal form was determined at a lower resolution, but using a construct with an authentic N terminus (PDB ID 6XR3). The structure discussed in the present study (PDB ID 7JKV) and the structure in 6XR3 nicely superimpose with an RMS 0.68 Å, and the substrate binding area is virtually the same. We also conclude that GRL-1720 and 5h completely block the infectivity and cytopathicity of SARS-CoV-2 by specifically targeting M^pro^ of SARS-CoV-2.

Our structural analyses suggest that both GRL-1720 and 5h form interactions with several active site residues, including Cys-145 of M^pro^. The catalytic dyad, His-41 and Cys-145, is involved in the nucleophilic attack on the ester carbon of GRL-1720, and the carbonyl indoline moiety is bound to Cys-145 of M^pro^ through a covalent bond (Supplementary Fig. 11). Indeed, the presence of a covalently-bound carbonyl indoline moiety was verified with nanoLC-ESI-QTOF-MS (Fig. 7). Notably, X-ray structural analysis revealed that the sulfur in Cys-145 of M^pro^ undergoes a nucleophilic addition reaction by forming a covalent bond with 5h (Fig. 6). However, it is also likely that 5h-M^pro^ covalently-linked ketal converts to a carbonyl group with non-covalent binding with M^pro^ in a reversible manner^35^. In fact, in our nanoLC-ESI-QTOF-MS studies, M^pro^ and 5h eluted separately, indicating that they do not form a covalent bond under the conditions used for our nanoLC-ESI-QTOF-MS studies. Overall, both the covalently bound tetrahedral ketal as well as the non-covalent bound carbonyl form of 5h likely make a number of interactions with the active site residues of M^pro^. 5h makes multiple additional favorable interactions with M^pro^ compared with GRL-1720, which may explain its much greater antiviral activity. Further, the thermal stability of M^pro^ significantly increased in the presence of 5h, corroborating that 5h also forms non-covalent bond with M^pro^ (Fig. 8).

Of note, the report by Jin et al.^24^ showed that (i) shikonin fits well to the hydrophobic cavity of the catalytic active site of M^pro^ in their docking simulation attempt and (ii) it moderately inhibited the enzymatic activity of M^pro^ in their enzyme assay. In our cell-based assays, however, shikonin showed no detectable antiviral activity against SARS-CoV-2 (Fig. 4). Most recently, a paper on the structure of M^pro^ complexed with shikonin was published by Li et al.^41^, showing that shikonin forms a non-covalent bond with M^pro^, although they did not report its actual antiviral activity against SARS-CoV-2. It is unknown as to whether further modification of shikonin can lead to the development of therapeutically useful compounds.

In conclusion, 5h, which shows potent antiviral activity and no significant detectable cytotoxicity, represents a promising lead compound to develop anti-SARS-CoV-2 agents. Moreover, the combination therapy using different class of agents, such as an M^pro^ inhibitor and an RdRp inhibitor, might be a promising therapeutic modality for treatment of SARS-CoV-2 infection.

## Methods

### Cells, viruses, and antiviral compounds

VeroE6 cells were obtained from Japanese Collection of Research Bioresources (JCRB) Cell Bank (Osaka, Japan) and were maintained in D-MEM supplemented with 10% FCS, 100 μg/ml of penicillin, and 50 μg/ml of kanamycin. Human peripheral blood mononuclear cells (PBMCs) and HBTEC were purchased from Lonza (Basel, Switzerland) and Lifeline Cell Technology (Frederick, MD), respectively. Calu-3 was kindly provided by Dr. Kawaoka (The University of Tokyo, Tokyo, Japan) SARS-CoV-2 JPN/TY/WK-521 strain (SARS-CoV-2^WK-521^) was obtained from National Institute of Infectious Diseases (Tokyo, Japan). Antiviral agents, GRL-1720 and compound 5h^20^ were synthesized and characterized (Supplementary Figs. 1 and 2) by Dr. Ghosh who is listed in co-author in this study, and shikonin was kindly provided by Dr. Hata (Kumamoto University, Kumamoto, Japan). Nelfinavir, atazanavir (Sigma-Aldrich, St. Louis, MO), and remdesivir (Selleck, Houston, TX) were purchased. All compounds were dissolved in DMSO at 20 mM concentrations as stock solutions.

### SARS-CoV-2 M^pro^ inhibition assays

SARS-CoV-2 M^pro^/3CL^pro^ (Accession #: MN908947) with the authentic N- and C-terminal residues that are released after cleavage from the polyprotein was used for all kinetic studies. The details for expression and purification of this fully active SARS-CoV-2 M^pro^ construct has recently been described^42^. In general, inhibition of SARS-CoV-2 M^pro^ by GRL-1720 and 5h was assessed using a continuous, fluorescence assay and the FRET-based substrate UIVT3 (HiLyte Fluor_488_^TM^–ESATLQSGLRKAK-QXL_520_^TM^-NH_2_) (Anaspec, Fremont, CA). The assay buffer consisted of 50 mM HEPES pH 7.50, 0.1 mg/mL BSA, 0.01% Triton X-100, 2 mM DTT, 1% DMSO and a final enzyme concentration of 200 nM. Kinetic assays were performed in Costar 3694 EIA/RIA 96-well half-area, flat bottom, black polystyrene plates (Corning, Corning, NY) at 25 °C. The increase in fluorescence intensity of SARS-CoV-2 M^pro^ catalyzed reactions was measured at an emission wavelength of 528 nm (20 nm bandwidth) using an excitation wavelength of 485 (bandwidth 20 nm) using either a CLARIOstar Plate Reader (BMG Labtech, Cary, NC) or a Synergy H1 hybrid multi-mode plate reader (BioTek, Winooski, VT). The initial rates of the reactions were then determined from the slopes of the Relative Fluorescence Units (RFU) produced during the initial rate period of the enzyme by time in minutes, yielding RFU min^−1^. Experimental details for the treatment of GRL-1720 kinetic data for irreversible covalent inhibition and 5h kinetic data for reversible covalent inhibition are provided in Supplementary information.

### Antiviral activity and cytotoxicity assay

For antiviral assay, cells were exposed to virus at multiplicity of infection (MOI) of 0.05 for 1 h, virus was then washed out, and cells were cultured in the presence or absence of compounds. After 3 days, cell culture supernatants were harvested and viral RNA was extracted using QIAamp Viral RNA Mini Kit (QIAGEN, Hilden, Germany), and quantitative RT-PCR (RT-qPCR) was then performed using One Step PrimeScript III RT-qPCR Mix (Takara Bio, Shiga, Japan) following manufactures’ instructions. The primers and probe used for detecting SARS-CoV-2 envelope^43^ were: 5′-ACT TCT TTT TCT TGC TTT CGT GGT-3′ (forward), 5′-GCA GCA GTA CGC ACA CAA TC-3′ (reverse), and 5′-FAM-CTA GTT ACA CTA GCC ATC CTT ACT GC-BHQ1-3′ (probe). Each assay was conducted in duplicate.

To determine cytotoxicity of a compound, VeroE6 and Calu-3 cells, and human primary cells, PHA-PBMC and HBTEC, were plated in a 96-well plate at a density of 10^4^ cells in 200 μL culture medium (final) in each well and were continuously exposed to various concentrations of the compound for 3 days. The cytotoxicity of compounds was determined using Cell Counting Kit-8 (Dojindo, Kumamoto, Japan). Each assay was conducted in duplicate.

In the experiments using airway epithelial cells, we used ALI culture model (EpiAirway; MatTeck, Ashland, MA). The maintenance medium with supplement (MatTeck) containing test compounds was added to ALI cultures (100 μL to the apical chamber and 500 μL to the basement chamber) and 100 μL of SARS-CoV-2^WK-521^ viral inoculum (2 × 10^4^ TCID_50_ per well) was added to the apical chamber. After 3 h of viral exposure, cells were washed and 500 μL of fresh maintenance medium containing the same concentration of the test compound was added to the basement chamber and the cells were cultured for 3 days. Immunocytochemistry and RNA-qPCR were performed after 3 days post viral exposure.

### Immunocytochemistry

Cells in 96-well microtiter culture plate were fixed with 4% paraformaldehyde in PBS for 15 min, washed with PBS (300 μL/well) three times for 5 min each, and were then blocked with a blocking buffer (10% goat serum, 1% BSA, 0.3% Triton X-100, and PBS 1X) for 1 h. After removing the blocking buffer, the cells were immediately stained with primary antibodies: convalescent IgG fraction (1/500 dilution), which was isolated from serum of a convalescent COVID-19 individual using spin column-based antibody purification kit (Cosmo Bio, Tokyo, Japan), overnight at 4 °C. The stained cells were washed with PBS (300 μL/well) three times for 5 min each, and the cells were incubated with the secondary antibody: goat polyclonal anti-human-IgG-Alexa Fluor 488 Fab fragment antibody (1/200 dilution)(Jackson ImmunoResearch Laboratories, Inc, West Grove, PA, USA), together with Texas Red^™^-X dye conjugated Phalloidin (Thermo Fisher Scientific, Waltham, MA, USA) for F-actin visualization for 2 h. After washing the cells with PBS (300 μL/well) three times for 5 min each, DAPI solution (Thermo Fisher Scientific) in PBS (50 μL/well) was added to stain nuclei. Signals were acquired with a Cytation 5 Cell Imaging Multi-Mode Reader and Gen 5 (v3.05) software.(BioTek).

### Analysis for combination effects

The Bliss additivism model^30–32^ was used to classify the effect of combining two agents as additive, synergistic, or antagonistic. A theoretical curve was calculated for combined inhibition using the equation bliss index (E_bliss_) = E_A_ + E_B_ − E_A_ × E_B_, where E_A_ and E_B_ are the fractional inhibitions obtained by drug A alone and drug B alone at specific concentrations. E_A+B_ is the fractional inhibition obtained by the combination with drug A and B. Here, E_bliss_ is the fractional inhibition that would be expected if the combination effect of the two drugs was exactly additive. If the experimentally measured fractional inhibition (E_A+B_) is less than E_bliss_, the combination effect is judged to be synergistic. If the experimentally measured fractional inhibition is greater than E_bliss_, the combination effect is judged to be antagonistic.

### Molecular modeling of the interaction of GRL-1720 with M^pro^

A crystal structure of SARS-CoV-2 main protease (M^pro^)(RCSB PDB ID 6Y2F) was used for molecular docking of GRL-1720. The crystal structure was processed for molecular docking by deleting the water molecules and dimethyl sulfoxide. Hydrogen atoms were added, and appropriate bond orders were assigned to the protein atoms, and the bound inhibitor. The protonation states of the asparagines, glutamines, and histidines were determined. Using the OPLS3 force field (used for all geometry minimizations), a restrained minimization was performed (with a cut-off of 0.30 Å for root mean square difference of heavy atoms from the crystal structure coordinates). The above were performed with the Protein Preparation wizard present in Maestro. The structure thus obtained was used for molecular docking. The inhibitor molecule GRL-1720 was built in Maestro, and minimized conformations were generated using the LigPrep module. The covalent docking sub-module of Glide was used and a docking grid encompassing the volume occupied by the bound inhibitor from the crystal structure was generated. A nucleophilic attack by Cys-145 of SARS-CoV2 protease on GRL-1720 was chosen as the mode of reaction while determining the docked interaction poses. This was because the literature on SARS-CoV and SARS-CoV-2 demonstrates that the active site cysteine undergoes nucleophilic addition reactions with formation of a covalent bond with appropriate functional groups such as a carbonyl carbon. Recent crystal structures also demonstrate that Cys-145 of SARS-CoV-2 protease forms covalent bond with inhibitors^24^. Subsequent to the formation of the covalent bond with M^pro^, GRL-1720, an ester, undergoes cleavage of the chloropyridinyl group forming a thiocarbonyl complex. The interactions of the thiocarbonyl complex with M^pro^ were determined by full geometry optimization. All simulations were done using software versions/modules as present in Maestro Version 10.7.015 (Schrödinger LLC, New York, NY).

### Expression and preparation of M^pro^

The SARS-CoV2 M^pro^-encoding sequence was cloned into pGEX-4T1 vector (Genscript, Piscataway, NJ). Prepared expression vector with GST tag was transformed to BL21-CodonPlus(DE3)-RIL strain (Agilent, Santa Clara, CA) by heat-shock transformation. The culture was grown in a shake flask containing 20 mL of LB medium plus ampicillin and chloramphenicol (LB^Am+^/^Cp+^) at 37 °C overnight. Twenty milliliter of the grown culture was added to 1 L of LB^Am+^/^Cp+^. The LB^Am+^/^Cp+^ culture was grown in flasks to an optical density of 0.5 at 600 nm at 37 °C, and the expression was induced by addition of 0.5 mM isopropyl β-D-thiogalactopyranoside for 6 h at 25 °C. After examination, the culture was spun down for pellet collection, and thus obtained pellets were stored at −80 °C until use. For purification of GST-linked M^pro^, each pellet was resuspended in PBS and lysed by sonication. The cell lysates were separated into a supernatant fraction and inclusion body fraction by centrifugation. The GST-tagged M^pro^ was confirmed to be present in the supernatant fraction. The GST-tagged M^pro^ was bound to 5 mL of Glutathione-Superflow Resin (Takara Bio). The resin was washed five times using 20 mL PBS. To cleave the GST tag form M^pro^, forty milliliter of PBS including 100 U of thrombin (GE Healthcare, Chicago, IL) was added to the resin and was incubated for 16 h at 25 °C. Following thrombin cleavage, intact M^pro^ with an additional N-terminal glycine residue was released from GST. Additional residue, Glysine does not influence to the structure/conformation of SARS-CoV-2 M^pro^. After incubation, GST-tag-cleaved M^pro^ was transferred to the supernatant form the resin. the GST-tag-cleaved M^pro^ was collected and diluted three times using buffer A (10 mM Tris pH7.5, 1 mM EDTA). The diluted M^pro^ was further purified by using HiTrap Q (GE Healthcare). The purified and concentrated SARS-CoV-2 M^pro^ (10–13 mg/mL) was stored in buffer A.

### Crystallization of M^pro^ and compound 5h

The M^pro^ was concentrated up to 3 mg/mL and incubated with 300 μM 5h for 1 h before crystallization. Crystals were grown using hanging drop vapour diffusion method at 20 °C. The reservoir solution contained 0.1 M MES pH 5.8, 15% polyethylene glycol (PEG) 6000, and 3% DMSO. Crystals were soaked briefly in a cryoprotection solution containing 0.1 M MES pH 6.0, 35% PEG 400 5% DMSO. X-ray data were collected at SPring-8 BL41XU (Hyōgo, Japan) and processed using DIALS using xia2 incorporated in ccp4i2^44^. The source wavelength for the data collection was 1.0 Å. Data collection statistics are shown in Supplementary Table S3. The phase problem was solved by molecular replacement using MolRep^45^ using the 2.16 Å structure of M^pro^ (PDB ID:6LU7) as a model. All water molecules and ligand atoms were omitted from the starting model. Subsequent cycles of refinement to 1.25 Å resolution were performed in REFMAC5^46^. Structure file of 5h was generated using the Dundee PRODRG2 server^47^ and manually fitted to the electron density. All structural figures were produced with PyMOL and UCSF Chimera^48^. The data were deposited into the PDB under ID: 7JKV.

### Electrospray ionization quadrupole time-of-flight mass spectrometry (ESI-QTOF/MS) analysis

To detect the molecular weight of the M^pro^, analysis was done using a quadrupole-time-of-flight (QTOF) mass spectrometer equipped with a Captive Spray electrospray ionization platform in the positive mode (impact II, Bruker Daltonics Bremen, Germany) with liquid chromatography (Ultimate 3000 HPLC, Thermo Fisher scientific). A sample (1 μl, 1 pmol) was separated on an Acclaim PepMap 100 C18LC column (0.075 mm × 150 mm, 2 μm particle) (Thermo Fisher scientific) over 35 min using a 2% acetonitrile gradient. Separations started with 95% buffer A, and 5% buffer B (acetonitrile with 0.1% formic acid) to 70% buffer B and then ramped to 95% in the following 5 min and then returned to starting conditions 5 min later. The intact and modified forms of M^pro^ eluted at ~27 min. Following ion source parameters have been applied: dry Heater: 150 °C, dry Gas: 8.0 L/min, capillary voltage: 1000 V, End plate offset: −500 V. MS scans have been acquired at a spectra rate of 1 Hz at a mass range from 100 to 3000 m/z. Molecular weights by protein deconvolution were determined using DataAnalysis 4.4 (Bruker Daltonics, Bremen, Germany).

### Thermal stability of M^pro^ in the absence or presence of 5h or lopinavir

Thermal stability was examined using the DSF. M^pro^ preparation (50 μM dissolved in 10 mM Tris (pH 7.5) and 1 mM EDTA was mixed with various amounts of a test compound and incubated at 37 °C for 3 h. Subsequently, 30 μL of the solution was successively heated from 15 °C to 95 °C, and the changes of fluorescence intensity were documented using the real-time PCR system (Applied Biosystems). The Tm (50% melting temperature) values were determined as the temperature at which the relative fluorescent intensity became 50%. Note that the thermal stability curves of M^pro^ with 5h at the molar ratios of 1:10 and 1:20 shifted to the higher temperature (to the right). The thermal stability curves of M^pro^ did not shift with lopinavir as compared with that with no agent.

### Reporting summary

Further information on research design is available in the Nature Research Reporting Summary linked to this article.

## Supplementary information

Supplementary Information

Reporting Summary

## Data Availability

Crystal structure data that support the findings of this study have been deposited in Protein Data Bank with the PDB ID: 6XR3, 7JKV. All relevant data supporting the findings in this study are available from the corresponding author upon reasonable request. Source data are provided with this paper.

## Source data

Source Data
