# Supplementary material for: A small molecule compound with an indole moiety inhibits the main protease of SARS-CoV-2 and blocks virus replication
Source: Nat Commun. 2021 Jan 28;12:668. doi: 10.1038/s41467-021-20900-6 (PMC7843602; doi:10.1038/s41467-021-20900-6)
Supplement: Supplementary file 1 — Supplementary Information [file 41467_2021_20900_MOESM1_ESM.pdf]

Supplementary Information

A small molecule compound with an indole moiety inhibits the main protease of SARS-CoV-2 and blocks virus replication

Shin-ichiro Hattori[1], Nobuyo Higashi-Kuwata[1], Hironori Hayashi[2,3], Srinivasa Rao Allu[4], Jakka Raghavaiah[4], Haydar Bulut[5], Debananda Das[5], Brandon J. Anson[6], Emma K. Lendy[6], Yuki Takamatsu[1], Nobutoki Takamune[7], Naoki Kishimoto[8], Kazutaka Murayama[9], Kazuya Hasegawa[10], Mi Li[11,12], David A. Davis[13], Eiichi N. Kodama[3,14], Robert Yarchoan[13], Alexander Wlodawer[11], Shogo Misumi[8], Andrew D. Mesecar[6], Arun K. Ghosh[4], and Hiroaki Mitsuya[1,5,15,*]

[1]Department of Refractory Viral Infections, National Center for Global Health and Medicine Research Institute, Tokyo, Japan; [2]Department of Intelligent Network for Infection Control, Tohoku University Hospital, Miyagi, Japan; [3]Department of infectious Diseases, International Research Institute of Disaster Science, Tohoku University, Miyagi, Japan; [4]Department of Chemistry and Department of Medicinal Chemistry and Molecular Pharmacology, Purdue University, West Lafayette, IN, USA; [5]Experimental Retrovirology Section, HIV and AIDS Malignancy Branch, National Cancer Institute, National Institutes of Health, Bethesda, MD, USA; [6]Department of Biochemistry and Department of Biological Sciences, Purdue University, West Lafayette, IN, USA; [7]Kumamoto Innovative Development Organization, Kumamoto University, Kumamoto, Japan; [8]Department of Environmental and Molecular Health Sciences, Faculty of Medical and Pharmaceutical Sciences, Kumamoto University, Kumamoto, Japan; [9]Graduate School of Biomedical Engineering, Tohoku University, Miyagi, Japan; [10]Protein Crystal Analysis Division, Japan Synchrotron Radiation Research Institute, Hyogo, Japan; [11]Protein Structure Section, Center for Structural Biology, National Cancer Institute, Frederick, MD, USA; [12]Basic Science Program, Leidos Biomedical Research, Frederick National Laboratory for Cancer Research, Frederick, MD, USA; [13]Viral Oncology Section, HIV and AIDS Malignancy Branch, National Cancer Institute, National Institutes of Health, Bethesda, MD, USA; [14]Department of Infectious Diseases, Graduate School of Medicine and Tohoku Medical Megabank Organization, Tohoku University, Miyagi, Japan; [15]Department of Clinical Sciences, Kumamoto University Hospital, Kumamoto, Japan

*Corresponding Author: hmitsuya@hosp.ncgm.go.jp

Contents:
Supplementary Table 1-3
Supplementary Figure 1-11
Supplementary Methods

Supplementary Tables

Supplementary Table 1. Cytotoxic profiles of 5h in human cell line and primary cells

Compounds	CC_{50} (μM)		
	Calu-3	PHA-PBMC[a]	HBTEC[b]
Remdesivir	>200	>200	138±9
5h	>200	>200	>200

CC_{50} values were determined with WST-8 assay and the assay was performed in duplicate. [a]Phytohemagglutinin (PHA)-stimulated peripheral blood mono nuclear cells (PBMC), [b]Human bronchial/tracheal epithelial cells (HBTEC).

Supplementary Table 2. Synergistic effect of 5h combined with remdesivr against SARS-CoV-2.

Conc. (μM)	Viral RNA (copies/ μL)				Bliss additivism values		
	None	Remdesivir (A)	5h (B)	Combined (A+B)	E_{bliss}	E_{A+B}	E_{bliss} / E_{A+B}
0	2.4×10^{10}						
1		2.3×10^{10}	2.4×10^{10}	1.9×10^{10}	0	14	0
2		3.4×10^{10}	1.8×10^{10}	1.3×10^{10}	20.3	43.7	0.464
4		4.3×10^{10}	1.1×10^{10}	9.5×10^{8}	49.4	95.8	0.515
10		1.2×10^{10}	1.0×10^{8}	3.8×10^{4}	99.8	100	0.998
20		7.7×10^{7}	1.1×10^{5}	1.4×10^{4}	100	100	1
40		5.1×10^{6}	3.6×10^{5}	1.9×10^{4}	100	100	1

The viral RNA copy numbers are shown as geometric means. The Bliss additivism method was utilized to define the synergism/antagonism of combinations of remedesivir and 5h. The effect of combination of the two drugs was determined as follows: $E_{bliss} / E_{A+B} = 1$ indicates that the combination effect was additive; $E_{bliss} / E_{A+B} < 1$ indicates that the percentage increase in maximal inhibition is above additivism (synergism); and $E_{bliss} / E_{A+B} > 1$ indicates that the percentage decrease in maximal inhibition is below additivism (antagonism). All assays were performed in duplicate and representative data from 2 independently conducted experiments are shown. Source data are provided as a Source Data file.

Supplementary Table 3. Data collection, phasing, and refinement statistics (molecular replacement)

	M^{pro}/5h (PDB ID: 7JKV)
**Data collection**	
Space group	P 1 21 1
Cell dimensions	
a, *b*, *c* (Å)	55.25, 99.14, 58.90
α, β, γ (°)	90, 107.86, 90
Resolution (Å)	56.06 – 1.25 (1.295 - 1.25) *
R_{sym} or R_{merge}	0.06192 (1.256)
I / σI	10.5 (1.0)
CC1/2	0.998(0.465)
Completeness (%)	95.2 (60.5)
Redundancy	6.1 (3.2)
**Refinement**	
Resolution (Å)	56.06 – 1.25
No. reflections (refined/free)	154907 / 3202
R_{work} / R_{free}	0.145, 0.177
No. atoms	5313
Protein	4727
Ligand/ion	98
Water	488
B-factors	26.31
Protein	25.28
Ligand/ion	23.31
Water	36.95
R.m.s deviations	
Bond lengths (Å)	0.013
Bond angles (°)	1.955

One crystal was used. *Values in parentheses are for highest-resolution shell.

Supplementary Figures

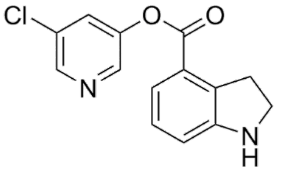

GRL-1720

Supplementary Figure 1. Structure and characterization of 5-chloropyridin-3-yl indoline-4-carboxylate (GRL-1720). ^{1}H NMR (400 MHz, Chloroform-*d*) δ 8.48 (d, *J* = 20.8 Hz, 2H), 7.67 (t, *J* = 2.1 Hz, 1H), 7.49 (dd, *J* = 7.9, 1.0 Hz, 1H), 7.15 (t, *J* = 7.8 Hz, 1H), 6.86 (dd, *J* = 7.8, 0.9 Hz, 1H), 3.65 (m, 2H), 3.45 (t, *J* = 8.5 Hz, 2H); ^{13}C NMR (101 MHz, $CDCl_3$) δ 164.3, 145.7, 141.4, 129.6, 127.7, 127.0, 124.4, 121.2, 120.5, 117.3, 114.3, 103.7, 46.8, 30.9; LRMS-ESI *(m/z)*: calcd for $C_{14}H_{11}ClN_2O_2$ 274.70; found 275.0 $[M+H]^+$; HPLC purity: 92% (YMC-PACK ODSA-A 250×4.6 mmI.D column, 40% $MeCN/H_2O$, 1.0mL/min, 254nm, 23° C, t_R = 23.6 min).

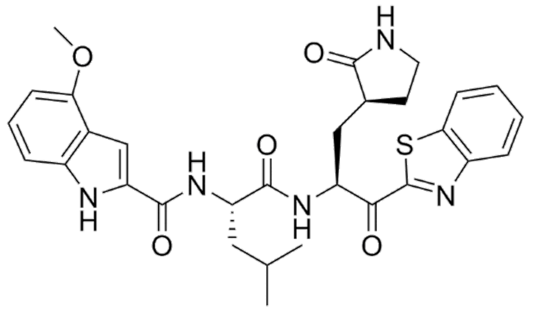

5h

Supplementary Figure 2. Structure and characterization of N-((S)-1-(((S)-1-(benzo[d]thiazol-2-yl)-1-oxo-3-((S)-2-oxopyrrolidin-3-yl)propan-2-yl)amino)-4-methyl-1-oxopentan-2-yl)-4-methoxy-1H-indole-2-carboxamide (5h). ^{1}H NMR (400 MHz, CDCl3) δ 9.70 (s, 1H), 8.59 (d, *J* = 6.5 Hz, 1H), 8.10 – 8.05 (m, 1H), 7.98 – 7.93 (m, 1H), 7.53 – 7.47 (m, 2H), 7.17 – 7.09 (m, 2H), 6.98 (d, *J* = 8.3 Hz, 2H), 6.46 (d, *J* = 7.7 Hz, 1H), 6.35 (s, 1H), 5.78 – 5.67 (m, 1H), 4.91 (td, *J* = 8.6, 5.3 Hz, 1H), 3.92 (s, 3H), 3.33 – 3.24 (m, 2H), 2.64 (dt, *J* = 16.1, 8.0 Hz, 1H), 2.53 – 2.42 (m, 1H), 2.27 – 2.20 (m, 1H), 2.15 (ddd, *J* = 14.0, 7.5, 3.4 Hz, 1H), 1.99 (dt, *J* = 19.5, 9.1 Hz, 1H), 1.86 – 1.77 (m, 2H), 1.72 – 1.64 (m, 1H), 0.96 (d, *J* = 5.7 Hz, 6H); 13C NMR (101 MHz, CDCl3) δ 192.0, 187.2, 179.8, 172.4, 163.9, 154.1, 153.3, 137.8, 137.1, 128.9, 127.8, 126.9, 125.5, 125.4, 122.3, 118.8, 105.0, 99.5, 55.2, 55.0, 51.6, 42.1, 40.5, 38.9, 33.0, 28.4, 24.7, 22.8, 22.1; LRMS-ESI *(m/z)*: calcd for $C_{30}H_{33}N_5O_5S$ 575.6; found 576.2 $[M+H]^+$; HPLC purity: 92% (YMC-PACK ODSA-A 250×4.6 mmI.D column, 50% MeCN/H_2O, 1.0 mL/min, 254nm, 23° C, t_R = 10.4 min).

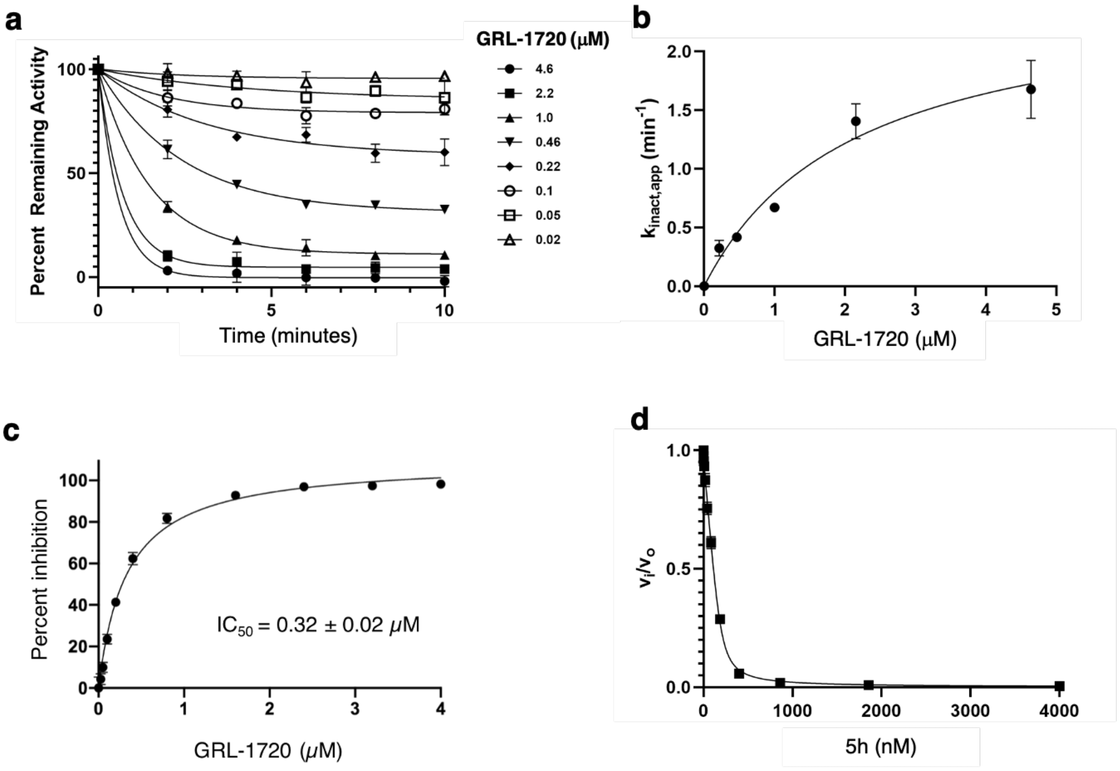

Supplementary Figure 3. Kinetics of inhibition of SARS-CoV-2 M^{pro} by GRL-1720 and 5h. The kinetics of irreversible inhibition of SARS-CoV-2 M^{pro} by GRL-1720 are shown in panels **a**-**c**. (**a**) Progress curves for the time-dependent inactivation of M^{pro} by increasing concentrations of GRL-1720 ranging from 0.02 to 4.6 μM. The data at each concentration shown were fit to Equation 1 to obtain the apparent rate constants for inactivation ($k_{inact,app}$). (**b**) The $k_{inact,app}$ values are plotted as a function of GRL-1720 concentration and the data were fit to Equation 2. The resulting kinetic parameters are kinact =2.53 ± 0.27 min^{-1} and K_i = 2.15 ± 0.49 μM. The second order rate constant k_{inact}/K_i = 19,610 M^{-1} ± 4,930 sec^{-1} (**c**). The kinetics of inhibition of SARS-CoV-2 M^{pro} by GRL-1720 after a 10 minute incubation of enzyme with increasing inhibitor concentrations and reaction initiation by the addition of substrate. Data were derived from Equation 4 and fit to Equation 5 and the resulting IC_{50} value is 0.32 ± 0.02 μM. (**d**) Kinetics of reversible-covalent inhibition of SARS-CoV-2 M^{pro} by 5h. A K_i value of 17.6 ± 3.2 nM was obtained from a fit of the data to the Morrison equation (Equation 6). The resulting fitted values for v_o and $[E]_t$ were 0.974 ± 0.006 and 194.8 ± 10.8 nM, respectively. The data shown with error bars in panels **a**, **b**, **c**, and **d** denote the mean values ± 1 S.D. of the assay results determined in triplicate (n=3). The data points accompanying no error bars had negligible 1 S.D. values (n=3).

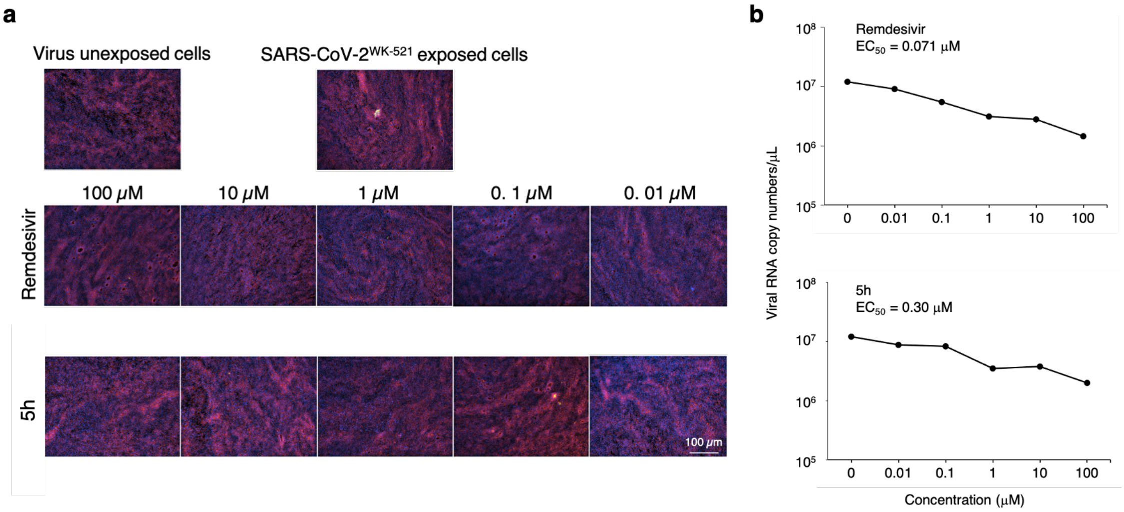

Supplementary Figure 4. Air-liquid interface (ALI) culture of human airway epithelial cells shows poor susceptibility to SARS-CoV-2^{WK-521} infection. (**a**) Immunocytochemistry images of cells cultured alone (top left inset), SARS-CoV-2^{WK-521}-exposed (2×10^4 $TCID_{50}$) cells cultured in the absence of compound (top right inset), virus-exposed and cultured in the presence of remdesivir (insets in the middle row) and 5h (insets in the bottom row) are shown. Note that human airway epithelial cells do not express detectable levels of SARS-CoV-2-related proteins, although the nature of those cells is of multilayer and it is possible that the antibodies did not stain infected cells. Representave images are shown from 2 indipendent experiments. (**b**) Viral RNA copy numbers in the culture supernatants in the presence of remdesivir (upper) and 5h (lower) and those with EC_{50} values are shown. Representative data from 2 indpendently conducted experiments are shown. The data points shown denote the mean values of the assay results determined in duplicate (n=2). Source data are provided as a Source Data file.

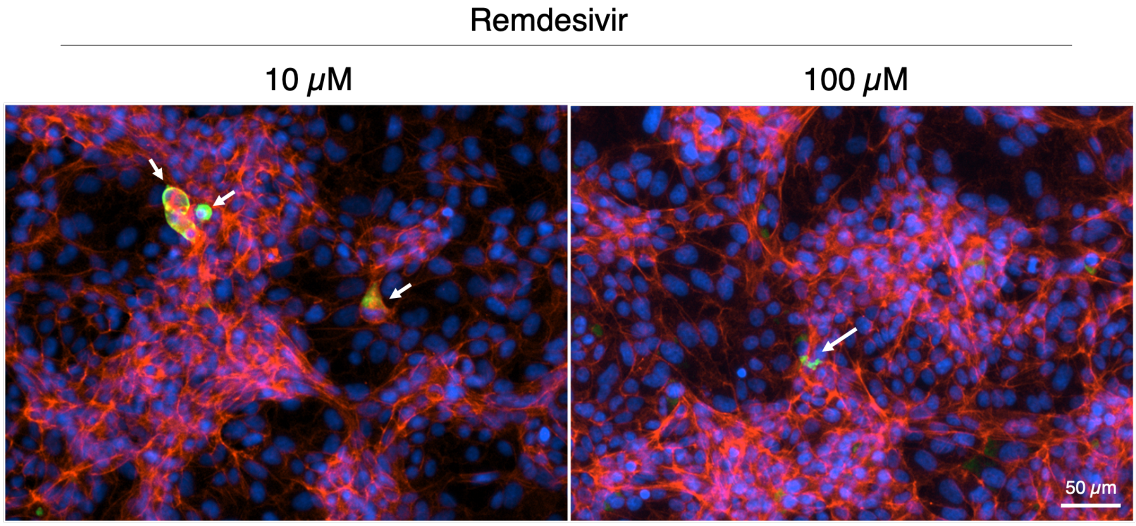

Supplementary Figure 5. Remdesivir permits breakthrough at high concentrations observed at high magnification. Although remdesivir potently blocks the infectivity and cytopathicity of SARS-CoV-2$^{WK\text{-}521}$, it allows viral breakthrough even at 100 μM (high magnification of Figure 4). Arrows denote the cells infected by the virus in the presence of high concentrations of remdesivir, representing virus breakthrough. Such breakthrough cells (stained in green) are often seen in clusters. The images shown here represent a part of a whole field, where infected cells are searched and identified, and do not represent the average of the whole field. Similar results were obtained in at least 10 independently conducted experiments.

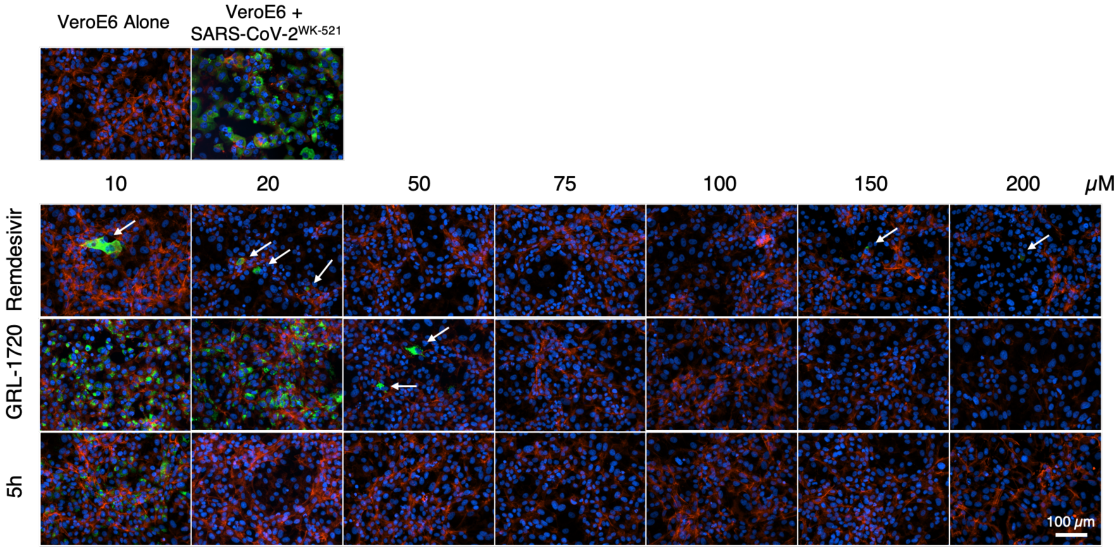

Supplementary Figure 6. GRL-1720 and 5h exert potent activity against SARS-CoV-2$^{WK\text{-}521}$ at a wide range of concentrations without significant cytotoxicity. VeroE6 cells were exposed to SARS-CoV-2$^{WK\text{-}521}$ for 1 hour, the virus was washed out, and the cells were cultured for 3 days in the presence of remdesivir, GRL-1720, or 5h. Immunocytochemistry was then performed using an IgG fraction from COVID-19 convalescent plasm as a primary antibody. SARS-CoV-2 antigens, F-actin, and nuclei are stained in green, red, and blue, respectively. In this set of the data, the infectivity of SARS-CoV-2$^{WK\text{-}521}$ was completely blocked by GRL-1720 at 75 μM and higher concentrations and by 5h at 20 μM and higher concentrations and no virus breakthrough was seen in the entire field of each well. However, in the cells cultured in the presence of remdesivir, virus breakthrough was identified even at high concentrations, 150 and 200 μM. Similar results were obtained in at least 5 independently conducted experiments.

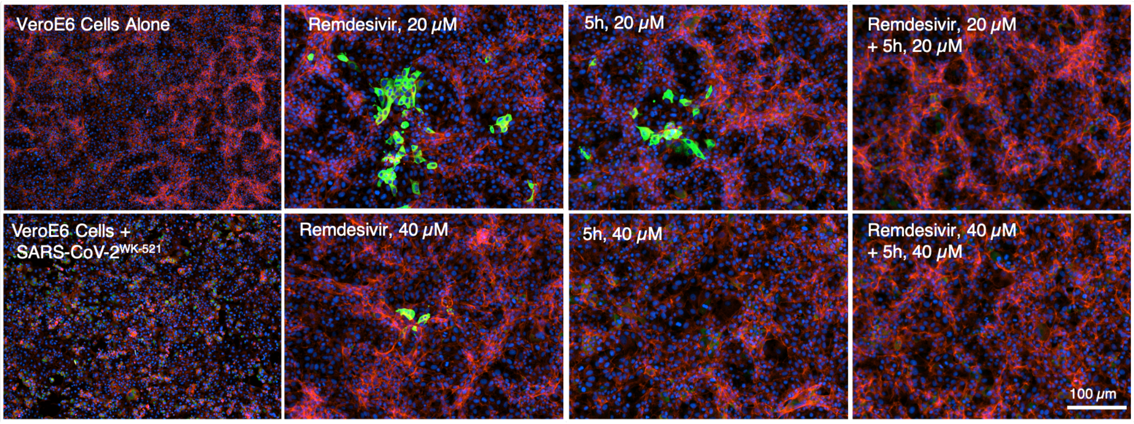

Supplementary Figure 7. Compound 5h combined with remdesivir exerts synergistic antiviral activity against SARS-CoV-2. Immunohistochemistry images of SARS-Cov-2$^{WK\text{-}521}$-exposed VeroE6 cells cultured in the presence of remdesivir alone, 5h alone, or remdesivir combined with 5h are shown. When cultured in the presence of remdesivir or 5h alone , there was significant virus breakthrough. However, when combined, there was no virus breakthrough at both 20 and 40 μM. SARS-CoV-2 antigens, F-actin, and nuclei are indicated in green, red, and blue, respectively. Similar results were obtained from 2 independently conducted experiments.

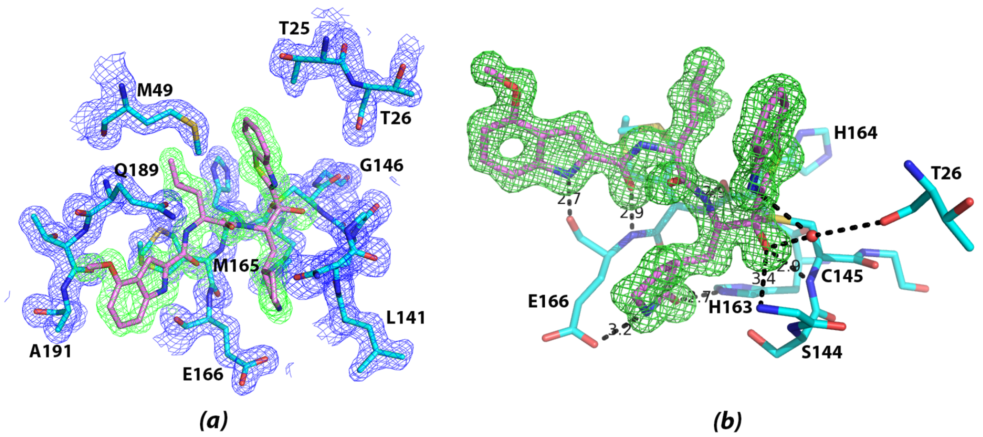

Supplementary Figure 8. The inhibitor and the surrounding residues of the protease with the relevant electron density. ***(a)*** A 2mFo-DFc electron density map showing 5h and the residues in the binding site of the protease. The model of 5h is colored purple and the map green, whereas the residues of the protease are colored cyan and the map blue. The map contour level is 1.0σ. ***(b***) An omit map calculated after refinement of the data excluding the inhibitor. Purple: 5h model; Cyan: residues interacting with 5h at the binding site; Green: mFo-DFc map contoured at 3σ level.

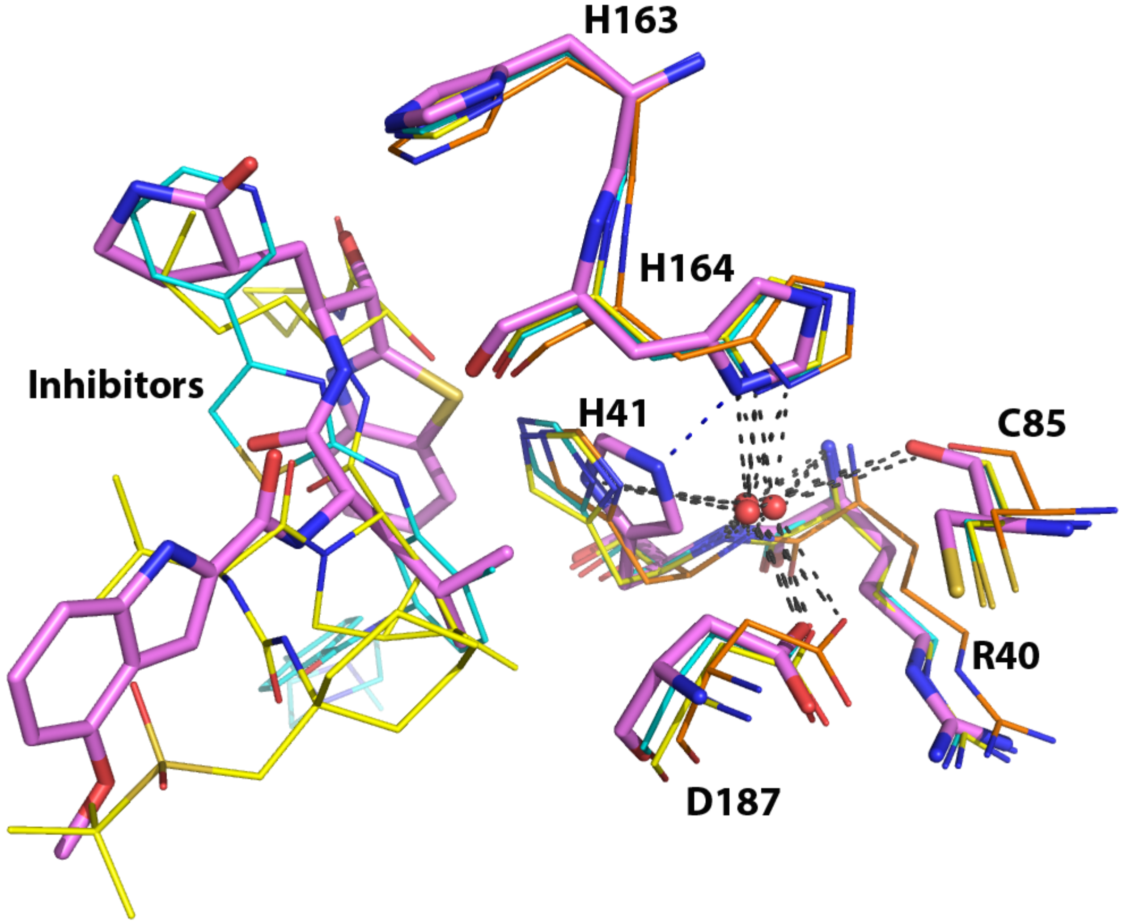

Supplementary Figure 9. The structures of the complex of the SARS-CoV-2 M^{pro} with inhibtors. A comparison of the structure of the complex of the SARS CoV-2 M^{pro} with 5h (purple) with the uninhibited structure of the enzyme (PDB ID 7c2y; orange), a covalent complex with narlaprevir (PDB ID 7jyc; yellow), and a non-covalent complex with masitinib (PDB ID 7ju7; cyan). Note that His41 has been pushed away from the locations seen in the other structures by direct interaction with the inhibitor. By making a hydrogen bond with His164 the side chain of His41 is occupying the area in the 5h complex where a water molecule is present in all other structures.

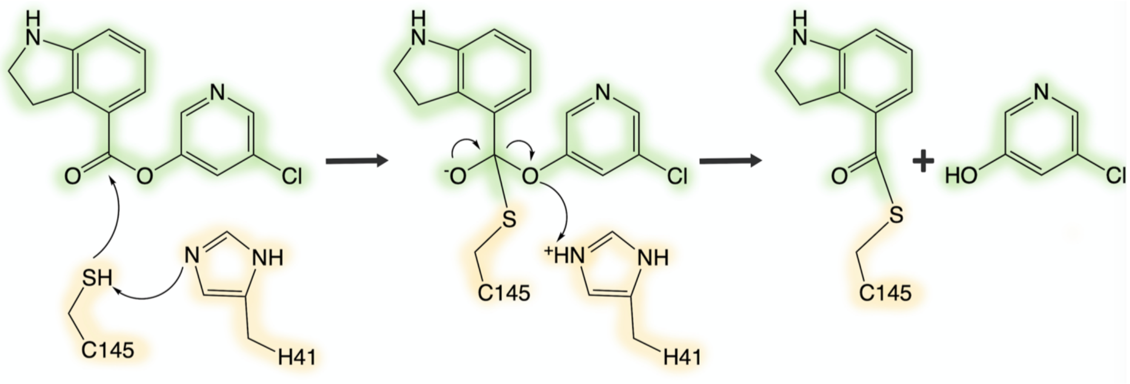

Supplementary Figure 10. The presumed mechanism of interactions of GRL-1720 with M^{pro}. In the interactions of GRL-1720, an indoline-chloropyridinyl ester (shaded in green), with M^{pro}, the catalytic dyad of M^{pro}, His41 and Cys145 (both shaded in yellow), are involved in the nucleophilic attack on the ester carbon of GRL-1720. Following acylation, the chloropyridinyl group departs, and the carbonyl indoline moiety is bound to Cys-145 of M^{pro} with a covalent bond. The presence of covalently-bound carbonyl indoline moiety was verified by ESI-QTOF/MS as shown in Figure 7.

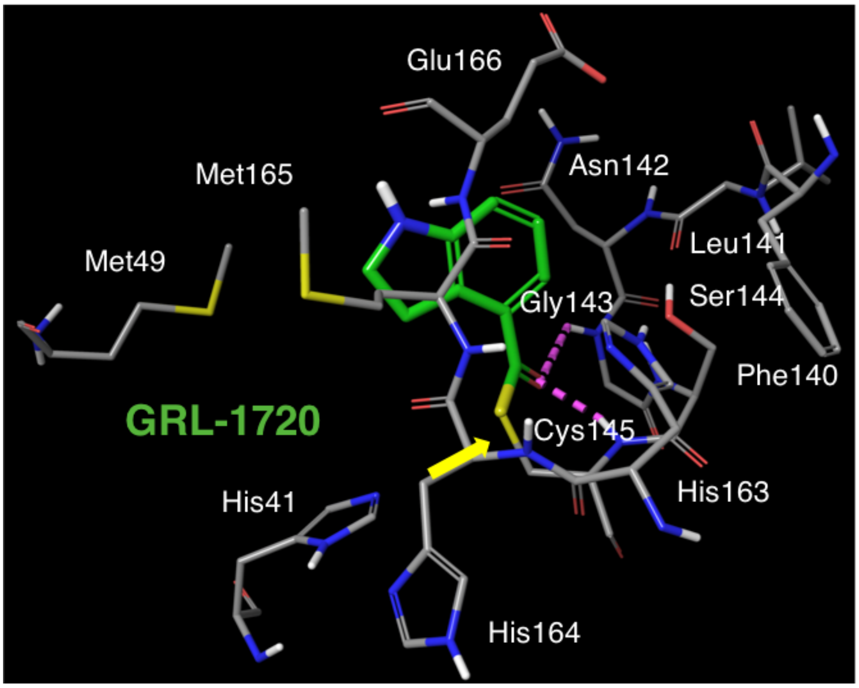

Supplementary Figure 11. Molecular model of the docked interactions of GRL-1720 with M^{pro}. GRL-1720 is shown in green, and selected M^{pro} residues in the active site are shown in gray. Only polar hydrogens are shown. Hydrogen-bond interactions are shown by magenta dotted lines. Nitrogen, oxygen, sulfur, and hydrogens are in blue, red, yellow, and white. The location of the covalent bond between GRL-1720 and Cys145 is shown by a yellow arrow. The figure was generated using Maestro Version 10.7.015 (Schrödinger LLC, New York, NY).

Supplementary Methods

Kinetics of Inhibition of SARS-CoV-2 M^{pro} by GRL-1720 and 5h.

GRL-1720: Since GRL-1720 acts as an irreversible inhibitor of SARS-CoV-2, the rates of inactivation of SARS-CoV-2 3CL^{pro} by GRL-1720 were assessed using the assay conditions described in the methods section. Both the enzyme and inhibitor (variable concentrations) were incubated in working stocks which were analyzed every two minutes over a total of ten minutes by taking 80 µL aliquots of these stocks and adding to 20 µL of the UIVT3 substrate. Fluorescence at each timepoint was measured in 20 s intervals using a Biotek Synergy H1 plate reader at an $\lambda_{excitation}$ of 485/20 nm and an $\lambda_{emission}$ of 528/20 nm for 80 s. The slopes (RFU/s) were converted to percent remaining activity by subtracting any background hydrolysis and dividing by the activity of the uninhibited enzyme. Final assay concentrations for each component are as follows: 50 mM HEPES pH 7.50, 0.1 mg/mL Bovine Serum Albumin, 0.01% Triton X-100, 2 mM DTT, 1% DMSO, 200 nM SARS-CoV-2 3CL^{pro}. GRL-1720 was serially diluted in DMSO utilizing a $10^{1/3}$ series, with final assay concentrations of: 4.64, 2.15, 1.00, 0.464, 0.215, 0.100, 0.0464, 0.0215, 0.0100, 0.00464, and 0 µM. The percent remaining activity (Y) values were plotted vs time (t) and the data were fit to a single-phase exponential decay model described by Equation 1.

Equation 1

$$Y = (\text{Percent Activity}_{\text{Max}} - \text{Percent Activity}_{\text{Inhibited}}) * e^{-k_{inact,app}*t} + \text{Percent Activity}_{\text{Inhibited}}$$

The apparent rate constants for inactivation, $k_{inact,app}$ for each concentration of GRL-1720 were obtained and then plotted against the compound's concentration, [I]. The data were then fit to Equation 2 to obtain the actual rate of inactivation, k_{inact}, at saturating inhibitor concentration, k_{inact}, and the K_i value for inhibition by GRL-1720.

Equation 2 $$k_{inact,app} = k_{inact} * \left(\frac{[I]}{K_i*[I]}\right)$$

The value for (k_{inact} / K_i) was then calculated along with its associated error σ(k_{inact} / K_i) which was calculated using Equation 3 and the associated error in the fitted parameters σk_{inact} and σK_i

Equation 3 $$\sigma\left(\frac{k_{inact}}{K_i}\right) = \left(\frac{k_{inact}}{K_i}\right) * \sqrt{\left(\frac{\sigma k_{inact}}{k_{inact}}\right)^2 + \left(\frac{\sigma K_i}{K_i}\right)^2}$$

An IC_{50} value for GRL-1720 was also determined. In a biochemical assay, the IC_{50} of an irreversible inhibitor will shift from a value that approximates the K_I to a value that correlates with the k_{inact}/K_I [1]. The rate of change over time will depend on the relationship between inhibitor concentration and rate of covalent modification defined by its k_{inact}/K_I. In a biochemical assay, the lower limit of the IC_{50} is one-half of the enzyme concentration. The IC_{50} value after a 10 minute incubation with the enzyme was therefore determined. One µL of 100x inhibitor stocks were prepared in DMSO and added to 79 µL of enzyme prepared in Assay Buffer prior to incubation at 25 °C for 10 minutes on an orbital shaker (Thermo Fisher Scientific). The reaction was initiated by adding 20 µL of the UIVT3 substrate followed by 10 s of vigorous shaking. The increase in fluorescence intensity of the reaction was measured with an $\lambda_{excitation}$ of 485/20 nm and an $\lambda_{emmission}$ of 528/20 nm for a period of 10 min. Relative Fluorescence Units (RFU) produced were plotted over time within the initial rate period of the reaction to yield RFU/s. The percent inhibition of SARS-CoV-2 M^{pro} by each inhibitor concentration was calculated using Equation 4 and these values were then fit to Equation 5 to determine the IC_{50} value.

Equation 4

$$\%\,Inhibition = 100\,\times\,[1 - \frac{Inhibited^{3CL^{pro}}\frac{RFU}{s} - Background\frac{RFU}{s}}{Uninhibited\ 3CL^{pro}\frac{RFU}{s} - Background\frac{RFU}{s}}]$$

Equation 5

$$\%\,Inhibition = \frac{\%I_{max} * [Inhibitor]}{IC_{50} + [Inhibitor]}$$

5h: In contrast to GRL-1720, 5h acts as a reversible-covalent inhibitor and therefore the kinetics of inhibition of SARS-CoV-2 were analyzed using reversible, tight-binding inhibition analysis. The assay conditions used are described in the methods section. The initial rates (RFU min^{-1}) at each inhibitor concentration were determined using a final enzyme concentration of 200 nM. Initial velocities were calculated by dividing the observed velocity in the absence of inhibitor (V_o) by the initial velocity at different inhibitor concentrations (V_i). The K_i value was then obtained by fitting the data to the Morrison equation (Equation 6) for tight-binding inhibitors similar to previously described methods [2].

Equation 6

$$\frac{V_i}{V_o} = \frac{[E]_o + [I]_o - K_i(1 + \frac{[S]}{K_m})}{2[E]_o} + \frac{\sqrt{([I]_o + K_i\left(1 + \frac{[S]}{K_m}\right) - [E]_o)^2 + 4[E]_o\,K_i\left(1 + \frac{[S]}{K_m}\right)}}{2[E]_o} \quad (3)$$

For these fits, the UIVT3 substrate concentration was fixed at a value of 2 μM and the K_m value was fixed at a value of 250 μM. The K_m value for the UIVT3 substrate is estimated to be even higher as even after correcting for inner-filter effects via dilution experiments, SARS-CoV-2 M^{pro} could not be saturated with substrate as the response of the enzyme to increasing UIVT3 substrate concentrations was linear over the range of 1 to 250 μM. The total SARS-CoV-2 M^{pro} enzyme concentration was experimentally determined by measuring the absorbance at 280 nm using the molar extinction coefficients ε_{280} calculated based on its primary amino acid sequence. The value for enzyme concentration $[E]_o$ in Equation 6 was allowed to float during the curve-fitting process. The resulting SARS-CoV-2 M^{pro} enzyme concentration from the Morrison equation fit was 195 ± 11 nM which is within error of the 200 nM SARS-CoV-2 determined experimentally and used in the assays.

References

1 Strelow, J. M. A Perspective on the Kinetics of Covalent and Irreversible Inhibition. *Slas Discov* **22**, 3-20, doi:10.1177/1087057116671509 (2017).

2 Yen, Y. C. *et al.* Development of an Efficient Enzyme Production and Structure-Based Discovery Platform for BACE1 Inhibitors. *Biochemistry-Us* **58**, 4424-4435, doi:10.1021/acs.biochem.9b00714 (2019).
