# Supplementary material for: A small molecule compound with an indole moiety inhibits the main protease of SARS-CoV-2 and blocks virus replication
Source: Nat Commun. 2021 Jan 28;12:668. doi: 10.1038/s41467-021-20900-6 (PMC7843602; doi:10.1038/s41467-021-20900-6)
Supplement: Supplementary file 2 — Reporting Summary [file 41467_2021_20900_MOESM2_ESM.pdf]

nature research

Corresponding author(s): Hiroaki Mitsuya

Last updated by author(s): Dec 15, 2020

Reporting Summary

Nature Research wishes to improve the reproducibility of the work that we publish. This form provides structure for consistency and transparency in reporting. For further information on Nature Research policies, see our Editorial Policies and the Editorial Policy Checklist.

Statistics

For all statistical analyses, confirm that the following items are present in the figure legend, table legend, main text, or Methods section.

n/a	Confirmed	
☐	☒	The exact sample size (*n*) for each experimental group/condition, given as a discrete number and unit of measurement
☒	☐	A statement on whether measurements were taken from distinct samples or whether the same sample was measured repeatedly
☒	☐	The statistical test(s) used AND whether they are one- or two-sided<br>*Only common tests should be described solely by name; describe more complex techniques in the Methods section.*
☒	☐	A description of all covariates tested
☒	☐	A description of any assumptions or corrections, such as tests of normality and adjustment for multiple comparisons
☐	☒	A full description of the statistical parameters including central tendency (e.g. means) or other basic estimates (e.g. regression coefficient) AND variation (e.g. standard deviation) or associated estimates of uncertainty (e.g. confidence intervals)
☒	☐	For null hypothesis testing, the test statistic (e.g. *F*, *t*, *r*) with confidence intervals, effect sizes, degrees of freedom and *P* value noted<br>*Give P values as exact values whenever suitable.*
☒	☐	For Bayesian analysis, information on the choice of priors and Markov chain Monte Carlo settings
☒	☐	For hierarchical and complex designs, identification of the appropriate level for tests and full reporting of outcomes
☒	☐	Estimates of effect sizes (e.g. Cohen's *d*, Pearson's *r*), indicating how they were calculated

Our web collection on statistics for biologists contains articles on many of the points above.

Software and code

Policy information about availability of computer code

Data collection	SPring-8 BL41XU; Gen5 (v3.05)
Data analysis	Graphpad Prism9 (v9.0.0); Xia2; Maestro (v10.7.015); REFMAC5; PyMOL (v1.3); UCSF Chimera; DataAnalysis 4.4; Microsoft Excel (v16.16.20)

For manuscripts utilizing custom algorithms or software that are central to the research but not yet described in published literature, software must be made available to editors and reviewers. We strongly encourage code deposition in a community repository (e.g. GitHub). See the Nature Research guidelines for submitting code & software for further information.

Data

Policy information about availability of data

All manuscripts must include a data availability statement. This statement should provide the following information, where applicable:

- Accession codes, unique identifiers, or web links for publicly available datasets
- A list of figures that have associated raw data
- A description of any restrictions on data availability

Crystal structure data that support the findings of this study have been deposited in Protein Data Bank with the PDB ID: 6LU7, 6XR3, 7JKV. All relevant data supporting the findings in this study are provided from the Source Data file or the corresponding author upon reasonable request.

Field-specific reporting

Life sciences study design

All studies must disclose on these points even when the disclosure is negative.

Sample size	Statistical methods were not used to determine sample sizes. However, for quantification purposes, a sample size of n = 2 unless otherwise stated (reproduced and confirmed by conducting the experiments on two or more independent occasions) was used. Sample sizes were determined based on prior experiences in the field.
Data exclusions	No data were excluded.
Replication	To ensure reproducibility of experimental findings, each assay was performed at least two times to confirm the results.
Randomization	Randomization for experiments was not relevant because all agents, antibody, and cells used for analysis were from the same initial stocks.
Blinding	Blinding for experiments was not relevant because all data collection and analysis were quantitative and not qualitative in nature.

Reporting for specific materials, systems and methods

We require information from authors about some types of materials, experimental systems and methods used in many studies. Here, indicate whether each material, system or method listed is relevant to your study. If you are not sure if a list item applies to your research, read the appropriate section before selecting a response.

Materials & experimental systems

n/a	Involved in the study	
☐	☒	Antibodies
☐	☒	Eukaryotic cell lines
☒	☐	Palaeontology and archaeology
☒	☐	Animals and other organisms
☒	☐	Human research participants
☒	☐	Clinical data
☒	☐	Dual use research of concern

Methods

n/a	Involved in the study	
☒	☐	ChIP-seq
☒	☐	Flow cytometry
☒	☐	MRI-based neuroimaging

Antibodies

Antibodies used	COVID-19 convalescent plasma-derived IgG (ConvIgG) was used as a primary antibody (1/500 dilution)(IgG was purified at National Center for Global Health and Medicine) and Alexa Fluor® 488 AffiniPure Fab Fragment Goat Anti-Human IgG (H+L) as a secondary antibody (1/200 dilution)(Jackson ImmunoResearch, 109-547-003) for immunostaining
Validation	SARS-CoV-2 infection and IgG amounts were determined with RNA-qPCR and ELISA, respectively. ConvIgG was validated using immunostaining of SARS-CoV-2-infected and -uninfected VeroE6 cells and the data obtained were confirmed to be free from non-specific detection.

Eukaryotic cell lines

Policy information about cell lines

Cell line source(s)	African green monkey origin, Vero cells from JCRB Cell Bank, Japan. Calu-3 cells from Dr. Yoshihiro Kawaoka (University of Tokyo, Japan)(ATCC, HTB-55).
Authentication	The authentication was performed by morphology check under microscopes.
Mycoplasma contamination	All cell lines used in the present study were tested negative for mycoplasma contamination.
Commonly misidentified lines (See ICLAC register)	No commonly misidentified cell lines were used.
